# Mechanisms of practice facilitation identified using system dynamics diagramming in a tailored implementation study of unhealthy alcohol screening and treatment in primary care

**DOI:** 10.1186/s43058-026-00909-y

**Published:** 2026-04-13

**Authors:** Erin S. Kenzie, Mellodie Seater, Chrystal Barnes, Tiff Weekley, Victoria Sanchez, Jennifer Coury, Brigit A. Hatch, Melinda M. Davis

**Affiliations:** 1 OHSU-PSU School of Public Health, Oregon Health & Science University, Portland, OR USA; 2 Oregon Rural Practice-Based Research Network, Oregon Health & Science University, Portland, OR USA; 3 Division of Oncological Sciences, Knight Cancer Institute, Oregon Health & Science University, Portland, OR USA; 4 Department of Family Medicine, Oregon Health & Science University, Portland, OR USA

**Keywords:** Practice facilitation, Quality improvement, Unhealthy alcohol use, System dynamics, Implementation mechanisms, Implementation strategies, Primary care

## Abstract

**Background:**

Practice facilitation is a valuable strategy for enhancing the adoption, implementation, and sustainability of evidence-based practices in primary care. It is important to understand how practice facilitation works to develop implementation strategies that are tailored to the needs of the practice. Research is needed to understand the mechanisms by which practice facilitation strategies impact clinic dynamics.

**Methods:**

We used a diagramming approach from qualitative system dynamics to model the mechanisms by which practice facilitators supported implementation in a pragmatic study of unhealthy alcohol use screening and treatment in primary care settings. The model was developed based on secondary analysis of qualitative data and participatory modeling sessions with study team members. We then analyzed the model to identify individual mechanisms underlying strategies used by practice facilitators by connecting strategies to feedback loops.

**Results:**

We produced two systems diagrams, one describing the dynamics of screening and treatment for unhealthy alcohol use in the usual care scenario and one illustrating how the practice facilitator strategies acted on those dynamics in the intervention. Facilitator strategies included health information technology support, workflow mapping, toolkit sharing, academic detailing, support finding community resources, goal setting, relationship building, needs assessment, and monthly facilitation sessions. We identified twenty-seven unique mechanisms connected with the facilitator strategies, which we illustrate using diagrams and narrative descriptions.

**Conclusions:**

Our analysis demonstrates that practice facilitation is a highly adaptive, context-sensitive meta-strategy. System dynamics diagramming is well suited for identifying and describing mechanisms of practice facilitation in implementation research because it illustrates how strategies act on existing dynamics to affect outcomes. This approach could provide a way to specify mechanisms of change in future implementation of screening and treatment of unhealthy alcohol use, as well as other evidence-based interventions.

**Trial registration:**

Not applicable.

**Supplementary Information:**

The online version contains supplementary material available at 10.1186/s43058-026-00909-y.

Contributions to the literature • The implementation science literature has called for approaches that account for complexity in implementation settings and specify implementation mechanisms, but existing methods are insufficient.

• By showing how strategies used by practice facilitators affect feedback dynamics in the existing context, system dynamics diagramming is well suited for illustrating implementation mechanisms.

• A definition of mechanisms based on system dynamics diagramming is provided that could be applied in future implementation research and practice.

• Practice facilitators can be considered ‘complexity navigators.

## Background

Practice facilitation is an effective strategy for supporting the implementation of evidence-based interventions in primary care settings [1]. Foundational literature offers a range of interpretations and approaches to practice facilitation [2–4]. Harvey and colleagues [2] suggested that the purpose of facilitation can be described as “ ranging from a discrete task-focused activity to a more holistic process of enabling individuals, teams and organizations to change.” Facilitators use a variety of context-specific skills and knowledge to influence change across professional and organizational boundaries to help health care providers and staff understand what they have to change and how they change it to increase the uptake of evidence into practice [4–6]. As such, practice facilitation has been also described as a “meta-strategy”, as facilitators may utilize, bundle, and/or tailor multiple implementation strategies [1, 7–11]. Interventions that address complex health issues have utilized practice facilitation to support quality improvement (QI) activities, or enhance the adoption, implementation, and sustainability of health programs or activities into practices [5, 10, 12–15].

Despite evidence of effectiveness, and an increased interest in using practice facilitation for implementation initiatives, little is known about the mechanisms underlying practice facilitation [16]. Moreover, within and across studies there is often varied impact on outcomes, as practice facilitation strategies themselves are diverse, depend on organizational context, and are often tailored by individual facilitators [2, 5, 7, 11, 17, 18]. Specifying these mechanisms could lead to more precise analysis and development of this meta-implementation strategy.

Methods to specify mechanisms of implementation strategies more broadly have been increasingly discussed within implementation science [19–24]. Mechanisms in this context have been defined as processes responsible for change [19, 25]. Specifying implementation mechanisms is important for understanding how, why, and under what conditions interventions succeed. Visualization of mechanisms (e.g., through causal pathway diagrams) has been recognized as important for clarifying the components of implementation mechanisms [16, 20, 26].

Systems science approaches, including system dynamics, have been increasingly used and called for in the implementation science literature to understand and manage complexity in implementation [22, 27, 28]. System dynamics is a systems science approach that uses diagrams and simulation models to identify a dynamic hypothesis describing how the causal structure of systems results in patterns of system behavior over time [29, 30]. This approach can emphasize the influence of idiosyncratic combinations of situation-specific factors in shaping outcomes [28, 30]. System dynamics has been applied widely in public health [30–37], including studies on implementing evidence-based interventions (EBIs) in clinical settings [38–40].

However, to our knowledge, no studies have applied methods from systems dynamics to identify mechanisms of practice facilitation. Because systems dynamics models visually describe feedback structures that explain how systems persist or change over time, it may be well suited for describing mechanisms of implementation strategies, such as practice facilitation [22, 27]. However, best practices for how to specify mechanisms using system dynamics diagramming notation have not been established.

Therefore, in this study we used system dynamics diagramming to identify the mechanisms underlying practice facilitation in Partnerships to Enhance Alcohol Screening, Treatment, and Intervention (ANTECEDENT), a pragmatic implementation study that used practice facilitation to support implementation of screening and treatment for unhealthy alcohol use (UAU) in primary care practices [31–33]. Prior research indicates that multiple strategies can be effective in improving implementation of screening, brief intervention, and referral to treatment (SBIRT) in primary care [34, 35]. Lounsbury and colleagues (2020) used system dynamics simulation modeling to compare the likely impacts of two SBIRT implementation strategies, continuous technical assistance and performance feedback reporting. To our knowledge, no studies have used this approach to identify mechanisms of practice facilitation. Our modeling will specify a dynamic hypothesis of usual care for SBIRT in primary care as well as a tailored intervention in which practice facilitators use strategies selected to meet clinic needs. We apply a definition of mechanisms based on model structure that could be applied to implementation contexts more broadly.

## Methods

We used a hybrid causal-loop/stock-and-flow diagramming approach from qualitative system dynamics to identify the mechanisms by which practice facilitators supported implementation of UAU screening and treatment in primary care settings in the ANTECEDENT study [31–33]. Our approach is based on a complex adaptive systems framework, which conceptualizes systems as being comprised of individual components or actors that interact to produce system behavior [38]. The diagram was developed based on secondary analysis of qualitative data and participatory modeling sessions with study team members. We then analyzed the diagram to identify individual mechanisms underlying strategies used by practice facilitators. Study activities were approved by the Oregon Health & Sciences University Institutional Review Board (IRB) through an expedited review (STUDY00020592).

### Study setting

ANTECEDENT was a pragmatic, flexible implementation study that used practice facilitation to support the implementation and integration of screening, brief intervention (BI), and referral to treatment (SBIRT) and medication-assisted treatment for alcohol use disorder (MAUD) in primary care clinics across the Pacific Northwest [31, 41].

In the study, practice facilitators provided highly tailored implementation support to primary care clinics. Facilitators were trained in QI approaches (e.g., plan-do-study-act [PDSA] cycles), SBIRT measure specifications, UAU, meeting management, and field note preparation. As detailed in the study protocol [31], all clinics participated in foundational support, consisting of a baseline assessment involving a clinic intake form, needs assessment call, health information technology (HIT) capacity assessment, and collection of SBIRT and MAUD performance data. They were also provided an SBIRT resource toolkit [42]. Clinics identified a primary point of contact for the study, most typically a clinic manager or clinician champion. Clinics could opt into a 15-month period of supplemental support, which included monthly practice facilitation sessions, as well as optional HIT expert consultation and support to enhance data reporting, academic detailing session(s) with an SBIRT motivational interviewing (MI) expert, data review (audit and feedback), and/or referral to peer-to-peer learning opportunities via the Oregon Extension for Community Health Outcomes (ECHO) Network or webinars. Facilitators were instructed to co-develop a tailored implementation plan with each of these clinics following the baseline assessment, and to support clinics in implementing this plan [33]. All meetings were shifted to virtual due to the COVID-19 pandemic.

Clinics were recruited between November 2019 and February 2022. Participating clinics were primary care practices willing to engage in quality improvement activities regarding SBIRT and MAUD and provide study data about implementation and patient outcomes. The first cohort of participating clinics began the intervention in February 2020, and the final cohort completed the intervention in April 2023. This timing was concurrent with the COVID-19 pandemic [43]. The ANTECEDENT intervention resulted in significant improvement in self-reported SBIRT outcomes and was found by clinics to be valuable [32, 37, 44–46].

### System dynamics

System dynamics is a field within systems science that uses diagramming and simulation modeling to facilitate understanding about how complex dynamics shape system behavior over time [30]. System dynamics diagrams and models describe dynamic hypotheses about how causal relationships between components of complex systems shape system outcomes [30]. As described by Sterman [30], a dynamic hypothesis is a theory about the feedback structure of a system that is believed to be responsible for its observed behavior. This hypothesis pertains to a specific problem or situation in a specific context. Researchers and decision-makers can use system models to generate useful insights into the system and examine anticipated or unanticipated effects of policies, interventions, or changes in context over time [47–49]. System models are also well suited for depicting continuous processes, capturing information flow, and illustrating feedback loops using causal-loop and/or stock-and-flow diagrams [43, 47, 50, 51]. Feedback loops are circular chains of causal relationships that drive nonlinear behavior in complex systems [30]. Reinforcing feedback loops describe exponential behavior, while balancing feedback loops describe tendency toward a set point or goal [30]. In a system dynamics approach, the behavior of a system is understood to be determined by its feedback structure, including the relative influence or strength of its loops [30].

In this study, we chose to use hybrid stock-and-flow/causal-loop diagramming, which includes stocks (places or states where patients accumulate), flows (ways for patients to move between stocks), auxiliary variables (contextual factors, strategies, and other informational variables), and causal links between variables, and feedback loops. This notation is described in more detail in prior work [30, 52]. This approach has been used in earlier research to show patient flow across health states or facilities [53–58]. Our approach used this hybrid diagramming notation to convey conceptual understanding of patient flows and did not involve any quantitative modeling or simulation. We chose to include stocks and flows in order to be able to specify places in which patients move through the UAU screening process, the factors that influence how they move through the system, and places that people could either get stuck or drop out. This hybrid diagram notation allows for this specificity without quantification of rates of accumulation or flow that would be found in a simulation model.

**Fig. 1:**
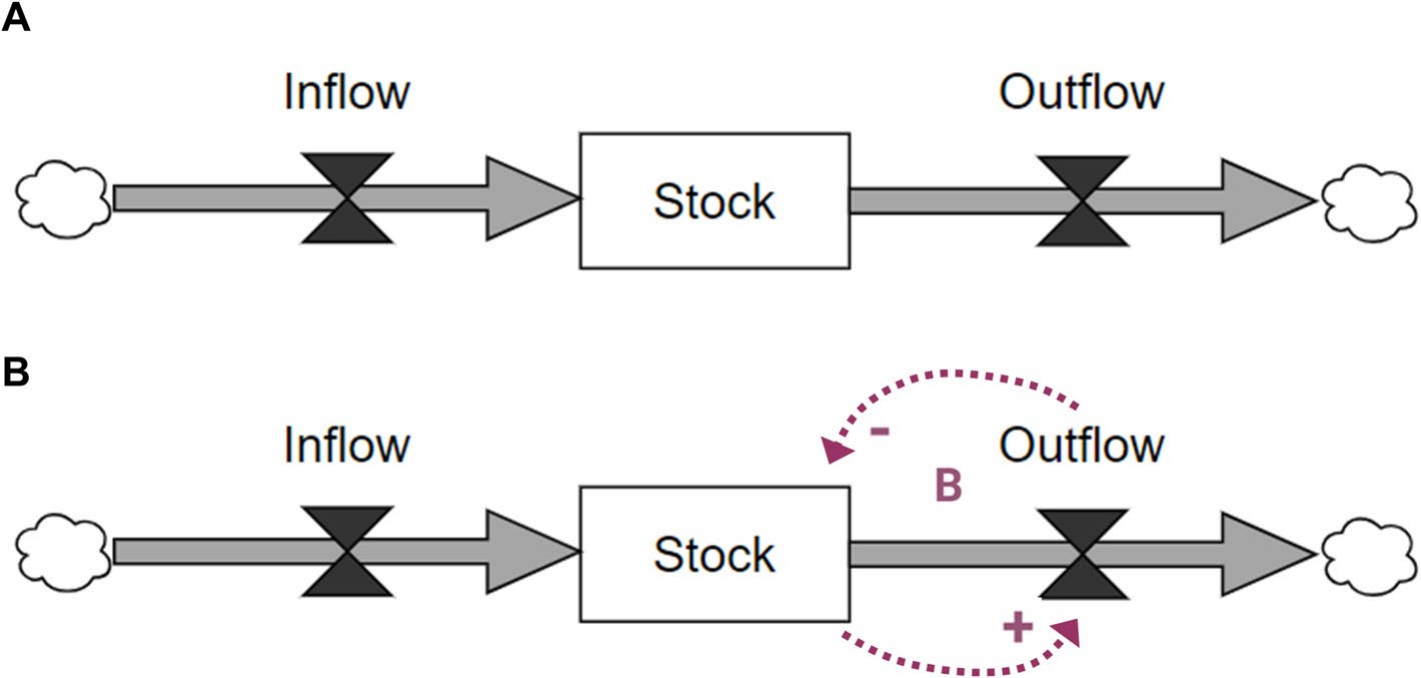
Implicit feedback dynamics in stock-and-flow diagrams. Figure 1**A** Shows a standard stock-and-flow diagram. The dotted lines in Fig. 1**B** Show the implicit feedback loop present in the outflow. The bottom arrow indicates that the greater the quantity of the stock, the greater the quantity of the outflow. The top arrow indicates that when the outflow increases, the stock decreases. This forms a balancing feedback loop (**B**). Figures generated using VisualParadigm Online and Microsoft Publisher

While a stock-and-flow diagram can appear linear, the compact notation contains implicit information about feedback dynamics. Specifically, any outflow implies an informational feedback communicating that the stock decreases as a result of the outflow. For example, suppose that a stock of ‘people needing to be screened’ for a certain medical condition is reduced via an outflow of ‘people being screened.’ The larger the outflow (i.e., the more people who receive screening), the fewer people remain in the stock of people needing screening. This implied causal link has a negative valence and creates a feedback loop with the causal link constituting the outflow. Figure 1 shows this hidden feedback structure in stock-and-flow diagrams.

### Participants

ANTECEDENT practice facilitators provided individualized, highly tailored implementation support to participating clinics based on their unique needs [31]. All facilitators were selected for this study because they had some experience in the fields of public health, substance use, and/or health services research. Nine practice facilitators were involved across the study’s active implementation period. The total length of participation varied by practice facilitator, ranging from 6–24 months.

### Data collection and modeling

We relied on two kinds of data for model development: secondary analysis of qualitative data collected as part of the ANTECEDENT evaluation and participatory modeling sessions with members of the study team. Sources of qualitative data used in secondary analysis include data collected from practice facilitators and clinics and are detailed in Table 1. Sample interview questions for qualitative data collection are included in Additional File 1 and are further detailed in the study protocol paper [31].

**Table 1:** Qualitative data sources for secondary analysis

Source	Collected by	Details
Clinic contact logs	Practice facilitators	Structured form and field notes tracked in REDCap immediately following clinic interactions
Practice facilitator interviews	Qualitative analyst interviewed facilitators	Conducted virtually every 6 months during implementation using semi-structured interview guide; transcripts validated against audio by research assistant
Periodic reflections	Qualitative analyst facilitated sessions with facilitators	Conducted virtually monthly during implementation; topics co-identified by analyst, facilitators and study team; transcripts validated against audio by research assistant
Clinic exit interviews	Qualitative analyst interviewed clinic representatives	Conducted virtually post-implementation using semi-structured interview guide; transcripts validated against audio by research assistant

All transcripts were uploaded into ATLAS.ti (version 23) for analysis. Transcripts were coded by two analysts (CB, TW) using a codebook developed for a comprehensive qualitative analysis of study data [31–33]. For this secondary analysis, we subcoded a subsection of the qualitative data initially coded as relating to implementation strategies, practice facilitator expertise, and facilitator mental models. Two analysts with expertise in system dynamics and qualitative analysis (MS, EK) developed and applied a secondary codebook based on practice facilitation strategies [6] and system dynamics model components [30, 52], detailed in Additional File 2. Coded data were then compiled in query reports and reviewed by the analysts in analytic discussions to identify causal structures to include in the models. An initial model was developed based on this analysis. The purpose of the model was to identify the mechanisms of practice facilitation in the ANTECEDENT study by specifying how strategies used by the facilitators acted on complex usual care dynamics. We chose to include dynamics within the primary care setting relating to SBIRT implementation and exclude other types of care and care provided outside the primary care setting.

Two participatory sessions with the study team provided an opportunity for model refinement. These sessions, which were based on Hovmand’s “modeling project community presentation” script [59], included practice facilitators, principal investigators, the project manager, research analysts, and research assistants. At the first session, the modelers (EK, MS) presented the draft models through a walkthrough and feedback was given by study team members. Participants were invited to consider variables that were missing, worded incorrectly, or could be deleted, as well as links between the variables. The modelers then revised the models based on this feedback. Proposed changes were discussed with the group until agreement was reached. A revised model was presented at a later session and revised based on group feedback into a final model. This iterative process of model development aligns with best practice in system dynamics [30]. Two diagrams were produced, one detailing the usual care scenario of SBIRT and MAUD implementation and one adding facilitator strategies used to support implementation in the ANTECEDENT study. Facilitator strategies, stocks, flows, and auxiliary variables were distinguished in the diagrams [30]. Kumu visualization software was used to produce model diagrams [60].

### Diagram analysis

While system dynamics is known as a mechanistic approach in that it explicates causal relationships between factors in a complex system, it does not contain guidance regarding the definition of individual mechanisms in the sense found in the implementation science literature. For the purposes of this analysis, we define a mechanism as any set of connected variables and causal links between a practice facilitation strategy and a feedback loop. Because outflows in stock-and-flow diagrams contain implicit feedback loops (see Fig. 1), this definition includes connections to feedback loops shown explicitly in the diagram as well as implicitly in the form of outflows.

After the diagram was completed, two analysts (EK, MS) applied the above definition to identify individual mechanisms. Mechanisms associated with each facilitator strategy were identified by tracking causal relationships from the strategy to flows or feedback loops. For strategies with multiple relationship pathways, each pathway was specified as an individual mechanism. Images of each mechanism were produced and described narratively.

## Results

To illustrate how strategies used by practice facilitators acted on existing clinic dynamics, we will present the model in several phases. The first diagram illustrates the usual care scenario of UAU screening and treatment prior to the ANTECEDENT study. The second diagram adds strategies used by practice facilitators. Finally, the mechanisms underlying these strategies as identified in the complete model will be described.

### Usual care model

**Fig. 2:**
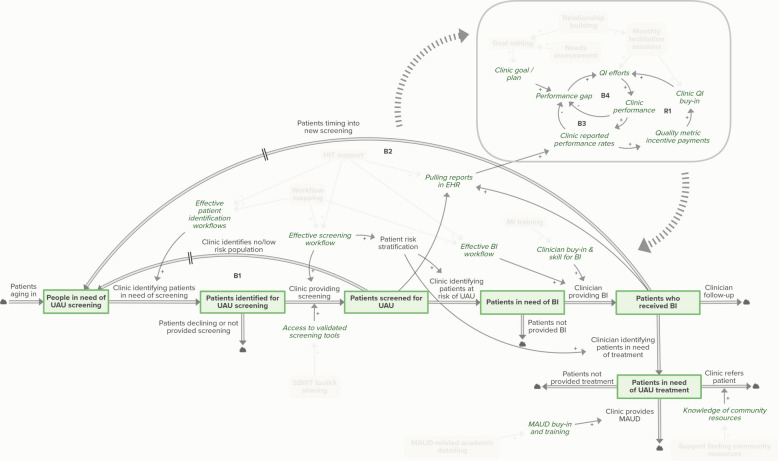
Usual care model for SBIRT and MAUD. Stock variables (green boxes) indicate points of accumulation; in this case, stocks are states of patient care. Thick double arrows between stocks constitute flows–ways in which patients move in or out of stocks. Cloud icons adjacent to flows show model boundaries. Green italics indicate additional auxiliary variables. Single arrows indicate causal links. A positive (+) valence indicates variables that increase or decrease in the same direction, while a negative (-) valence indicates variables that move in opposite directions. Large dashed arrows indicate interaction between submodel and patient flow model. Balancing (B) and reinforcing (R) feedback loops are numbered. Abbreviations: BI = brief intervention, EHR = electronic health record, MAUD = medication for alcohol use disorder, QI = quality improvement, UAU = unhealthy alcohol use

The usual care diagram shown in Fig. 2 illustrates key variables and causal links pertaining to screening and treatment under SBIRT and MAUD best practice, as understood by the ANTECEDENT study team and based on qualitative study data gathered from practice facilitators and clinics. The flow structure of the diagram shows how patients move through phases of screening and treatment. Patients in need of UAU screening are identified for screening and screened. Patients identified as having risky levels of alcohol use receive brief intervention and some are provided MAUD in clinic or are referred out for treatment. Feedback loops B1 and B2 show how patients time into new screening following screening or provision of brief intervention. Points at which patients drop out of this system (e.g., *Patients not provided BI*) are also included. A submodel in the upper right of the diagram shows how clinic QI efforts are supported by goal development/planning, tracking of performance rates, and incentive payments provided by Medicaid health plans.

### Facilitation model

**Fig. 3:**
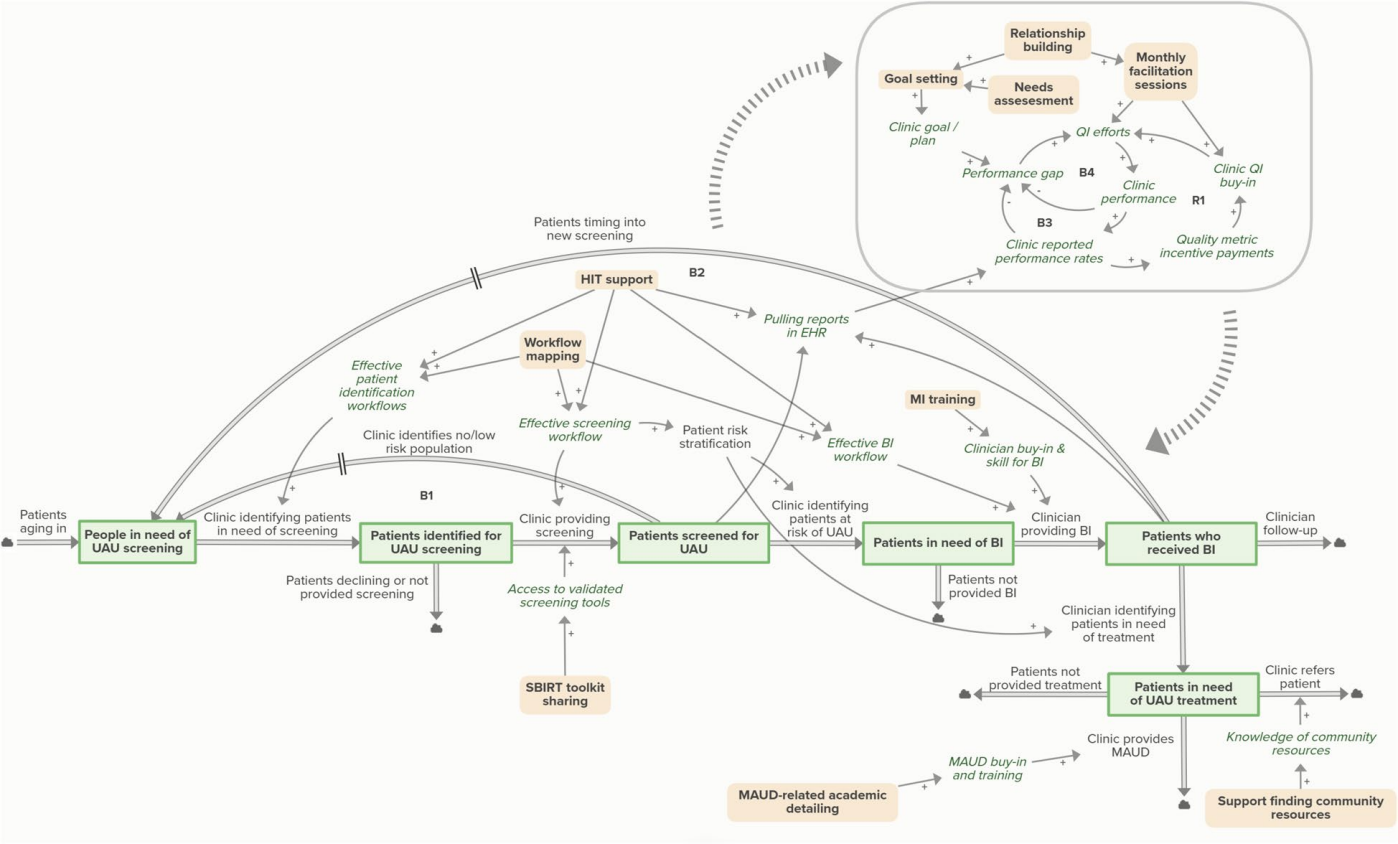
Model of facilitation mechanisms in ANTECEDENT study. Peach-colored variables with bold text indicate practice facilitation strategies. Stock variables (green boxes) indicate points of accumulation; in this case, stocks are states of patient care. Thick double arrows between stocks constitute flows–ways in which patients move in or out of stocks. Cloud icons adjacent to flows show model boundaries. Green italics indicate additional auxiliary variables. Single arrows indicate causal links. A positive (+) valence indicates variables that increase or decrease in the same direction, while a negative (-) valence indicates variables that move in opposite directions. Large dashed arrows indicate interaction between submodel and patient flow model. Balancing (B) and reinforcing (R) feedback loops are numbered. Abbreviations: BI = brief intervention, EHR = electronic health record, MAUD = medication for alcohol use disorder, QI = quality improvement, UAU = unhealthy alcohol use

The full model in Fig. 3 illustrates how strategies used by practice facilitators act on variables in the usual care model. Strategies include health information technology (HIT) support, workflow mapping, SBIRT toolkit sharing, MAUD-related academic detailing, support finding community resources, goal setting, relationship building, needs assessment, and monthly facilitation sessions. Table 2 below provides an outline of the operational definitions for each practice facilitation strategy.

**Table 2:** Practice facilitation strategies and operational definitions

Strategy	Definition
Goal setting	Collaborative development of implementation goals that are closely aligned with the unique context, needs, priorities, and preferences of the clinic and in accordance with study aims ([1]; Kenzie et al. 2025)
HIT support	Use of data experts to help improve clinic’s health information technology and monitoring systems by facilitating structured data entry, data extraction from electronic health record systems, and addressing technical issues impacting data quality and validity [1, 6, 31, 61]
MAUD-related academic detailing	Use of faculty expert consultations and educational outreach to improve provider knowledge regarding medications for alcohol use disorder and support standardization of prescribing practices ([1, 31, 37])
MI training	Use of expert consultations and educational outreach to improve provider skill, confidence, and consistency in conducting motivational interviewing ([1]; Kenzie et al. 2025)
Monthly facilitation	A meta-strategy and continuous process that supports clinic quality improvement. It involves setting improvement targets, monitoring progress, building capacity, solving problems, and guiding the implementation of study resources. The aim is to address identified needs for improvement and cultivate strong relationships among the study team, experts, and clinic partners [1, 31]
Needs assessment	A process designed to orient clinic partners to the study and formally capture each clinic's organizational culture, external context, motivation for participating in the project, current practices around unhealthy alcohol use, barriers and facilitators to implementation, as well as prior experience with facilitation and QI projects [1, 31]
Relationship building	An ongoing process of building trust, cultivating relationships with clinic partners, engaging leadership, and facilitating the development of academic partnerships for the purposes of shared training and bringing research skills to support the implementation work of clinical partners [1, 6, 61]
SBIRT toolkit sharing	The distribution of educational materials on the SBIRT Oregon website to support SBIRT implementation and improvement [1, 31]
Support finding community resources	The process of identifying and sharing local resources for patients in need of treatment for alcohol use disorders with clinic partners (Kenzie et al. 2025)
Workflow mapping	Documentation and diagramming of clinical workflows to understand clinic processes related to SBIRT and MAUD and the adjustment of workflows to build clinical capacity and create efficiencies by making explicit the roles and responsibilities of clinical staff in the provision of SBIRT [6, 31, 61]

### Practice facilitation mechanisms

Table 3 details the implementation strategies used by practice facilitators (referred to as practice facilitation strategies or facilitation strategies) and 33 mechanisms by which these strategies were understood to affect implementation and outcomes. Each of these mechanisms specifies a pathway from a facilitation strategy to a feedback loop in the model shown in Fig. 3. The mechanisms either help patients move through a specific step in the SBIRT process or support clinic-level improvement processes that in turn support SBIRT implementation. Most of the mechanisms, including the ones connected to outflows, include a balancing feedback loop. Several of the mechanisms include the R1 loop related to incentive payments and clinic buy-in. Many of the mechanisms are similar to each other, either because the same loops are strengthened or because the strategies activate similar pathways. We include each individual mechanism here to provide a comprehensive overview of distinct mechanisms.

**Table 3:** Practice facilitation strategies and mechanisms

Strategy	Mechanism(s)	Description
Goal setting	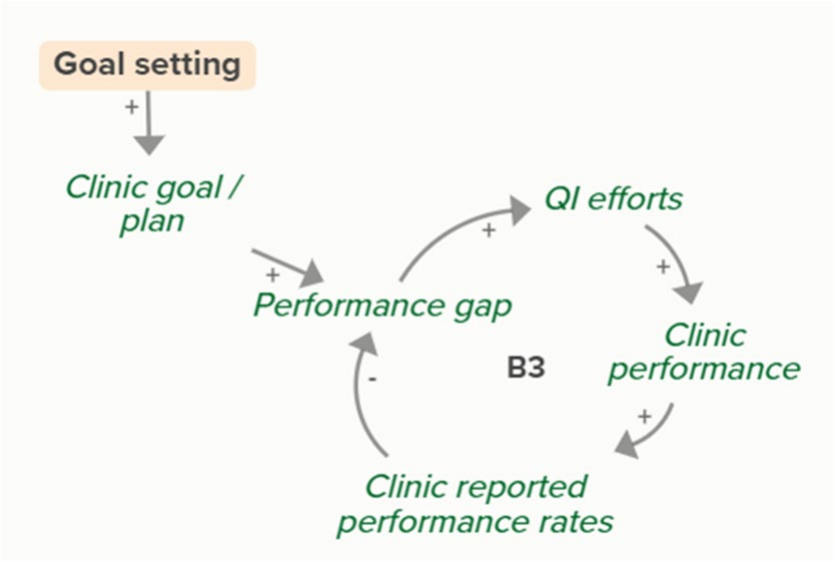	Facilitators utilized goal setting as a strategy for co-creating tailored implementation plans with participating clinics. When the goal exceeds current clinic performance, tension is created in a performance gap that motivates QI efforts to improve performance, which improves the clinic’s reported performance rates and reduces the performance gap. B3 is a goal-directed balancing feedback loop based on reported performance
	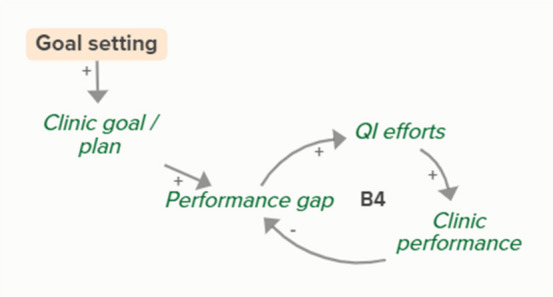	Facilitators utilized goal setting as a strategy for co-creating tailored implementation plans with participating clinics. When the goal exceeds current clinic performance, tension is created in a performance gap that motivates QI efforts to improve performance, which reduces the performance gap. B4 is a goal-directed balancing feedback loop that refers to performance beyond reported rates
	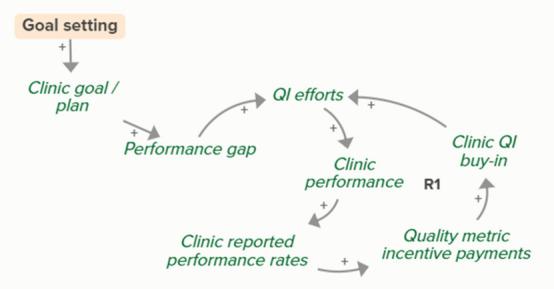	Goal setting supports the development of a clinic goal/plan. The performance gap prompts QI efforts, which improve clinic performance and reported improvement rates. R1 depicts a reinforcing feedback loop in which quality metric incentive payments increase clinic QI buy-in, which results in further strengthening QI efforts (R1 loop)
HIT support	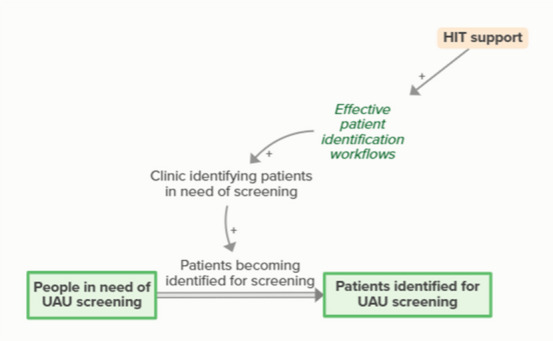	HIT support helps the development of effective patient identification workflows, which supports clinic identification of patients in need of screening. When clinics identify patients in need of screening, people in need of screening become patients identified for screening. As more people become identified for UAU screening, fewer people are in need of UAU screening, forming an implicit balancing feedback loop
	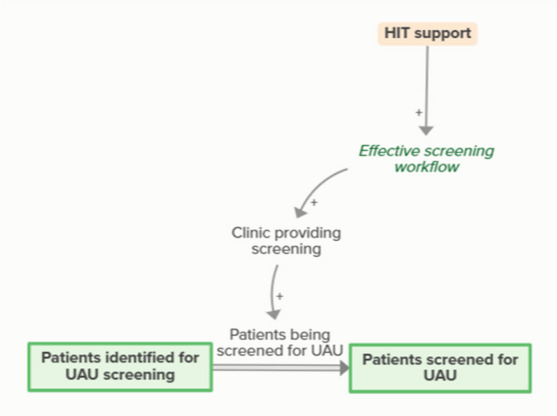	HIT support improves effective screening workflows, which are necessary for clinics to provide screening. When clinics provide screening, patients become screened and patients identified for screening become patients screened for UAU. As more patients are screened for UAU, fewer patients are identified for future UAU screening, forming an implicit feedback loop
	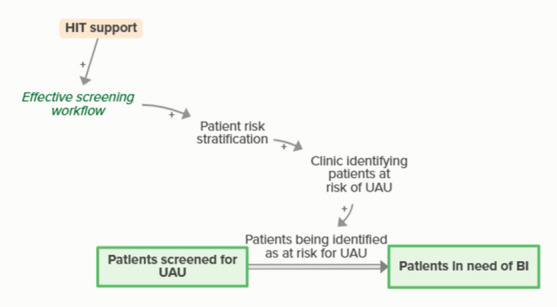	HIT support improves effective screening workflows, which facilitate the clinic’s ability to identify and stratify patients’ risk for UAU. As more patients are screened and identified as at risk for UAU, the number of patients in need of BI increases and the number of patients screened for UAU but not yet identified as in need of BI decreases, forming an implicit feedback loop
	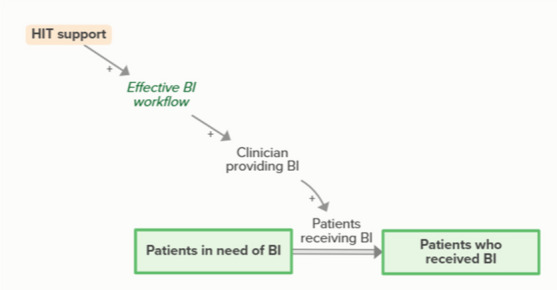	HIT support improves effective BI workflows, which facilitate clinicians providing BI. When clinicians provide and patients receive BI, patients in need of BI become patients who received BI. As more patients receive BI, fewer patients are in need of BI, forming an implicit feedback loop
	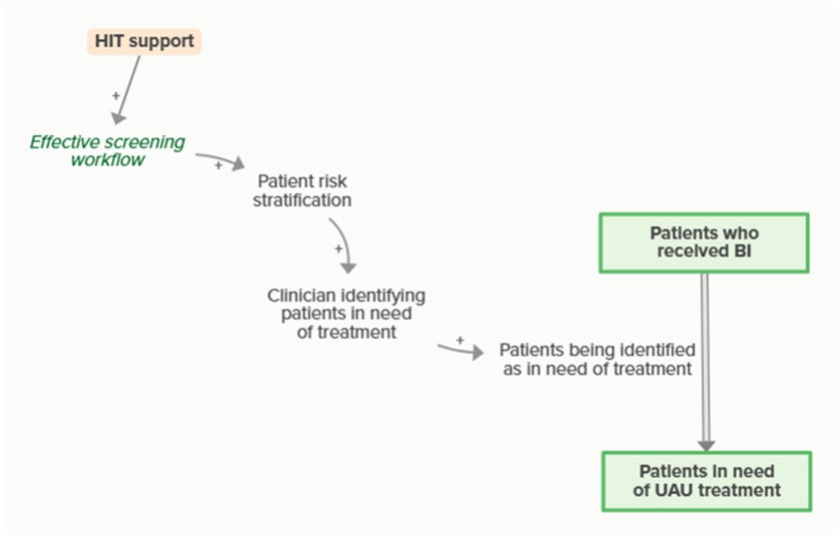	HIT support improves effective screening workflows, which facilitates the clinic’s ability to identify and stratify patients’ risk for UAU and need for more intensive UAU treatment. As clinicians identify patients needing UAU treatment, more patients are identified, meaning that some people who receive BI are transferred to a stock of people waiting for more intensive treatment. This outflow creates an implicit balancing feedback loop.
	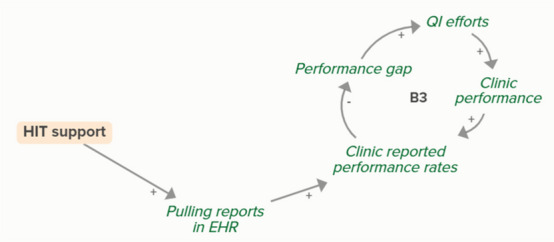	HIT support can improve a clinic’s ability to pull reports in their EHR, which is necessary for identifying clinic reported performance rates. Loop B3 describes how learning about performance rates changes the performance gap, which prompts QI efforts and improves clinic performance, ultimately circling back to reported performance rates (B3 loop)
	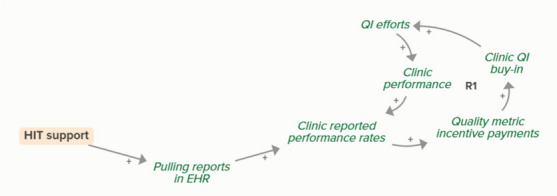	HIT support can improve a clinic’s ability to pull reports in their EHR, which is necessary for identifying clinic reported performance rates. Increased reported performance rates results in quality metric incentive payments, which increases clinic buy-in and QI efforts, ultimately resulting in improved performance and reported performance (R1 loop)
MAUD-related academic detailing	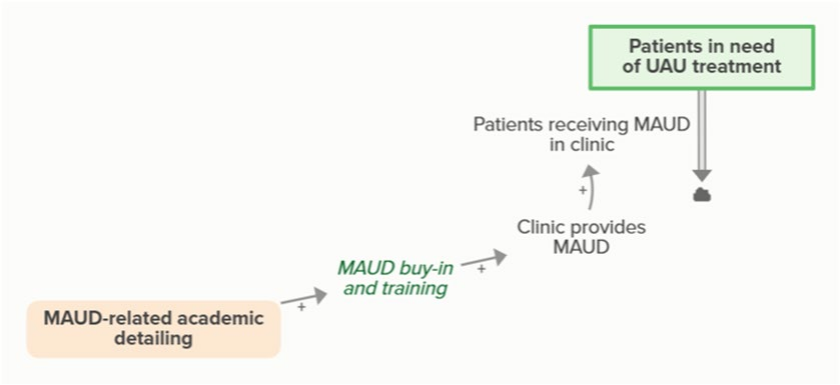	Facilitators arrange for faculty to provide academic detailing to clinicians regarding MAUD, which increases MAUD buy-in and training, ultimately increasing the likelihood that a clinic will provide and patients will receive MAUD. When patients receive MAUD, fewer patients are in need of UAU treatment, forming an implicit feedback loop
MI training	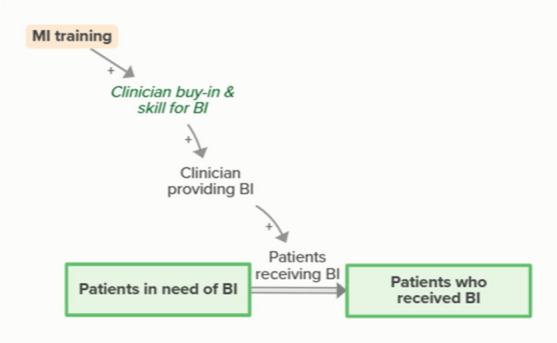	Motivational interviewing (MI) training increases clinician buy-in and skill for BI, which supports clinicians providing BI. When clinicians provide and patients receive BI, patients in need of BI become patients who received BI. As more patients receive BI, fewer patients are in need of BI, forming an implicit feedback loop
Monthly facilitation	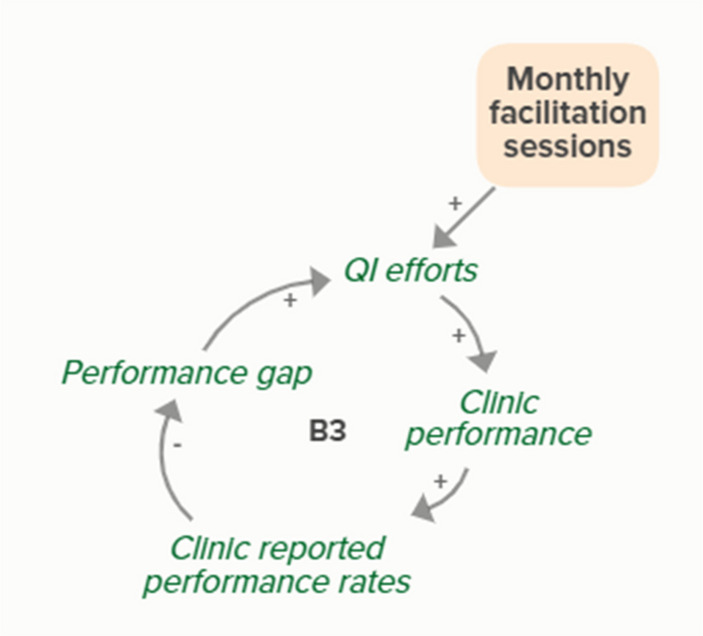	Monthly facilitation sessions support clinics’ QI efforts, which support improvement of clinics’ reported performance rates over time through the B3 balancing feedback loop
	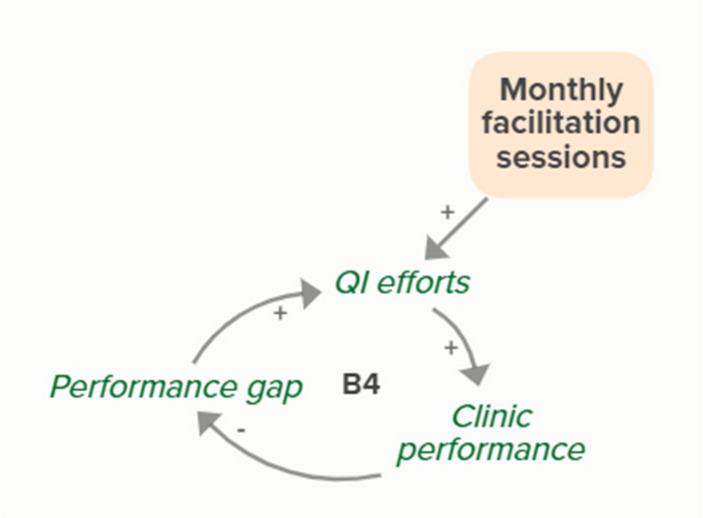	Monthly facilitation sessions support clinics’ QI efforts, which support improvement of clinics’ performance over time through the B4 balancing feedback loop
	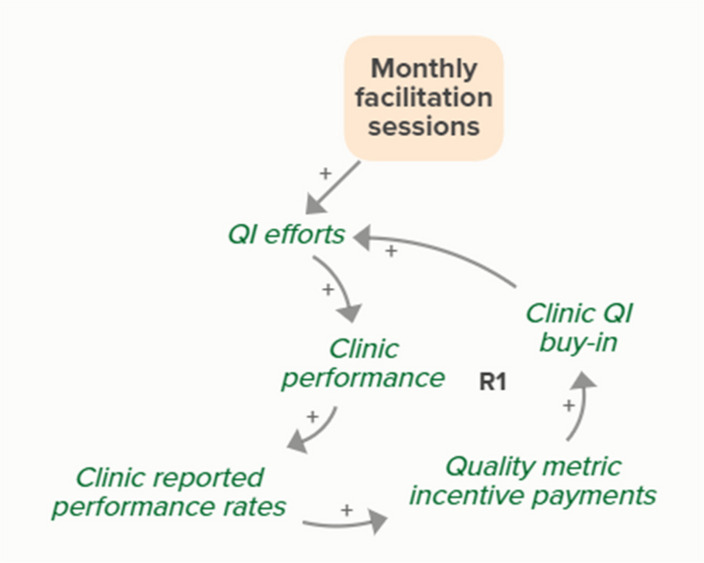	Monthly facilitation sessions support clinics’ QI efforts, which improve clinic performance and therefore clinic reported performance rates. Higher reported performance rate leads to quality metric incentive payments, which increases clinic QI buy-in, ultimately strengthening QI efforts (R1 Loop)
	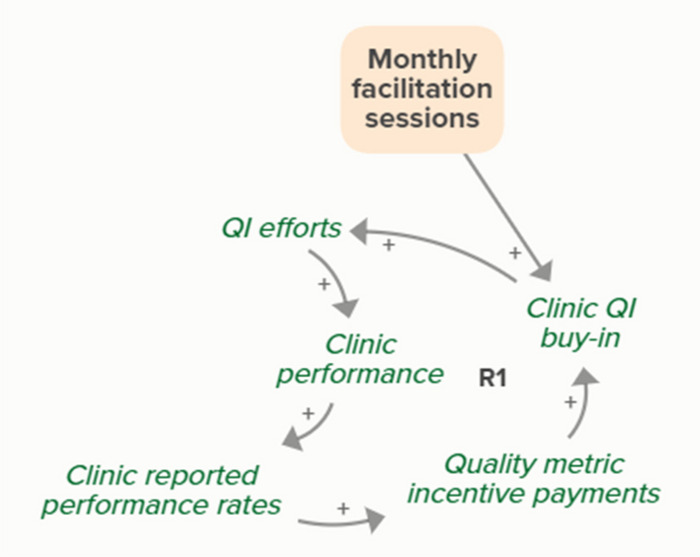	Monthly facilitation sessions support improved clinic QI buy-in, which feeds into the R1 loop that describes increased QI efforts and buy-in over time from the clinics receiving payment from improved performance. Increased reported performance rates results in quality metric incentive payments, which increases clinic buy-in and QI efforts, ultimately resulting in improved performance (R1 Loop)
Needs assessment	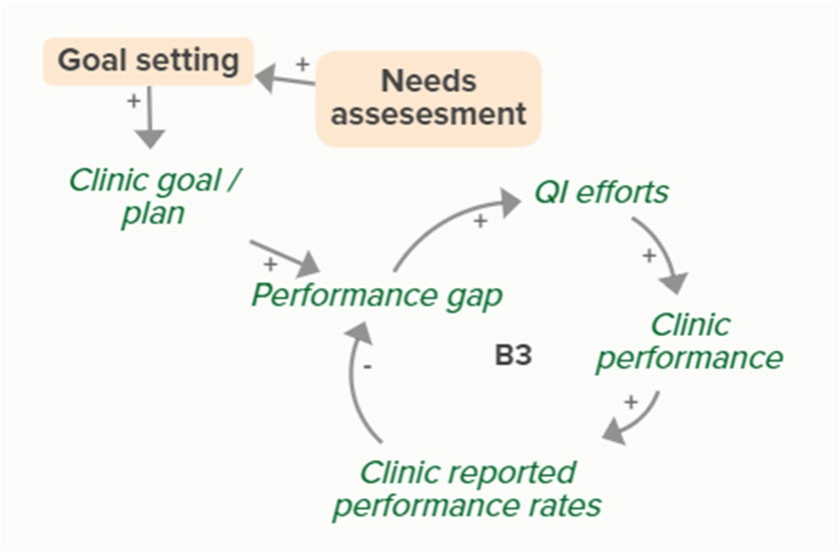	The needs assessment conducted by the practice facilitators informed goal setting and supported the development of a clinic goal/plan. A comparison between this goal and clinic reported performance rates forms the basis of the B3 loop that indicates increased QI efforts in the case of a performance gap
	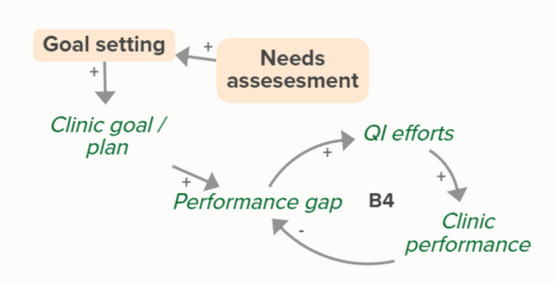	The needs assessment conducted by the practice facilitators informed goal setting and supported the development of a clinic goal/plan. When the goal exceeds current clinic performance, tension is created that motivates QI efforts to improve performance, which reduces the performance gap. B4 is a goal-directed balancing feedback loop that refers to performance beyond reported rates
	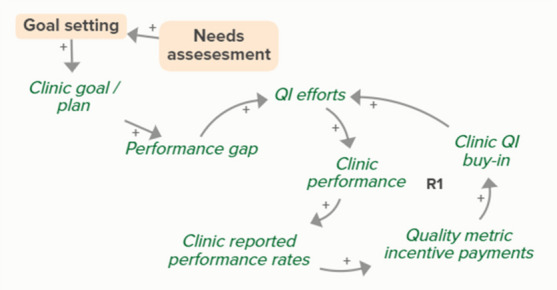	The needs assessment conducted by the practice facilitators informs goal setting and supports the development of a clinic goal/plan. The performance gap prompts QI efforts, which improve clinic performance and reported improvement rates. As performance improves, quality metric incentive payments increase and promote clinic QI buy-in, which results in further strengthening of QI efforts and subsequent clinic reported performance (R1 Loop)
Relationship building	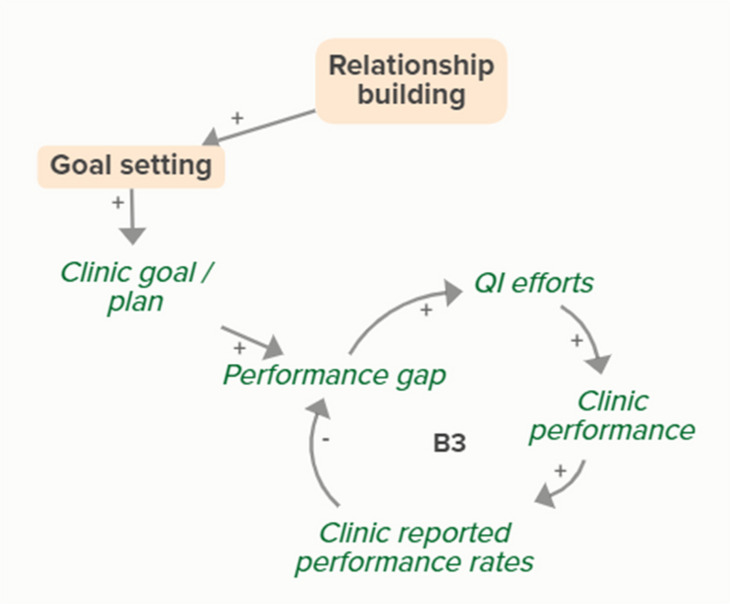	Relationship building supports goal setting, which supports the development of a clinic goal/plan. This goal supports the goal-directed feedback loop (B3) of QI efforts improving clinic performance over time based on reported performance rates
	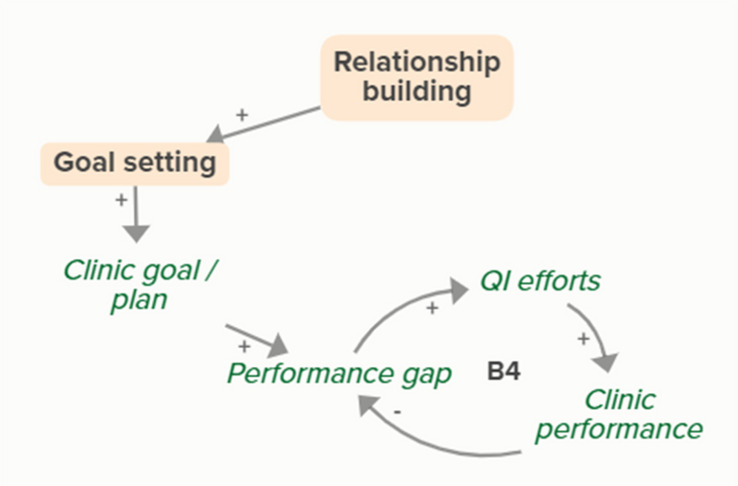	Relationship building supports goal setting, which supports the development of a clinic goal/plan. This goal supports the goal-directed feedback loop (B4) of QI efforts improving clinic performance over time
	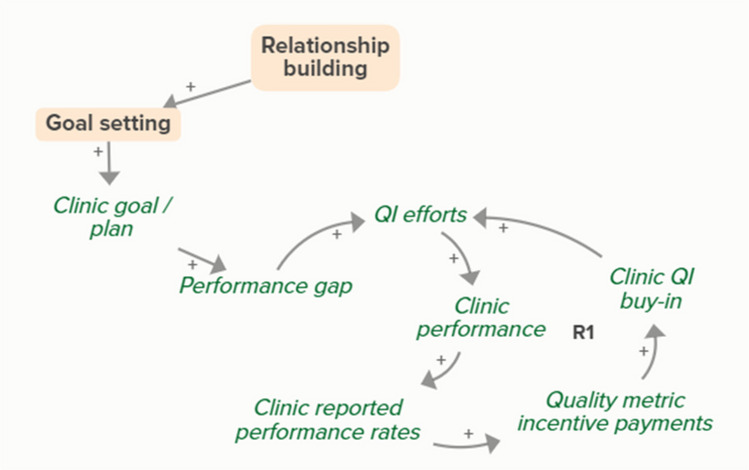	Relationship building supports goal setting, which supports the development of a clinic goal/plan. The performance gap prompts QI efforts, which improve clinic performance and reported improvement rates. Quality metric incentive payments increase clinic QI buy-in, which result in further strengthening QI efforts (R1 Loop)
	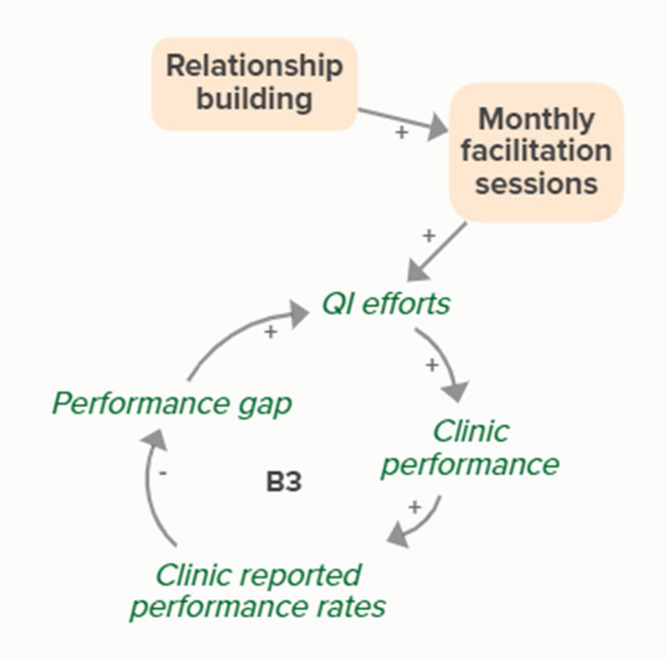	Relationship building strengthens monthly facilitation sessions, which supports QI efforts and the associated feedback loop to improve clinic performance based on reported performance rates
	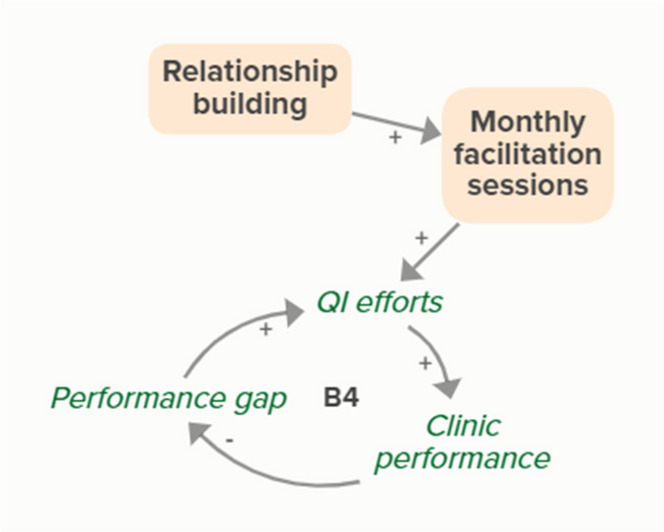	Relationship building strengthens monthly facilitation sessions, which supports QI efforts and the associated feedback loop to improve clinic performance
	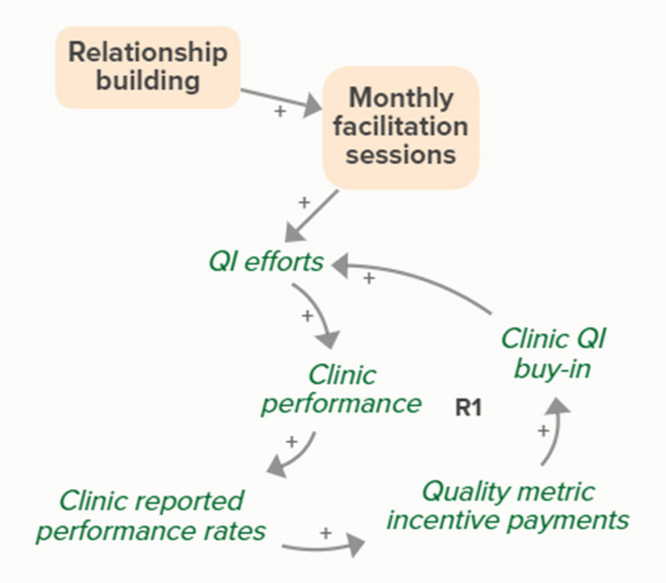	Relationship building strengthens monthly facilitation sessions, which supports QI efforts. Improved QI efforts support improved clinic performance and greater buy-in for QI based on quality metric incentive payments (R1 Loop)
	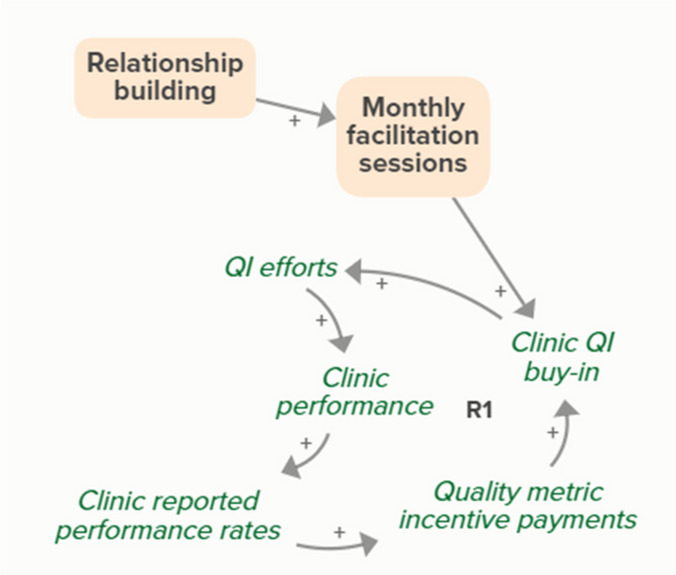	Relationship building strengthens monthly facilitation sessions, which improves clinic QI buy-in. Improved clinic QI buy-in results in greater QI efforts and buy-in based on quality metric incentive payments (R1 Loop)
SBIRT toolkit sharing	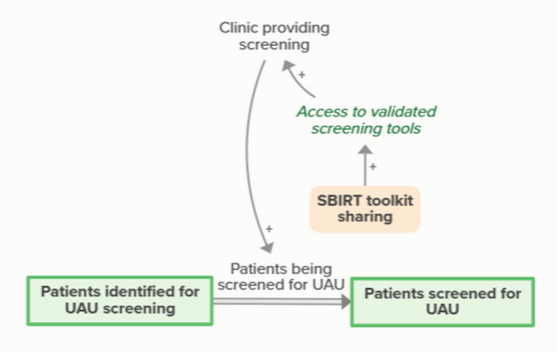	Sharing of the SBIRT toolkit enables access to validated screening tools, which supports clinics’ ability to provide screening. Provision of screening means that patients who are identified for UAU screening become screened. As more patients are screened for UAU, fewer patients are identified for futureUAU screening, forming an implicit feedback loop
Support finding community resources	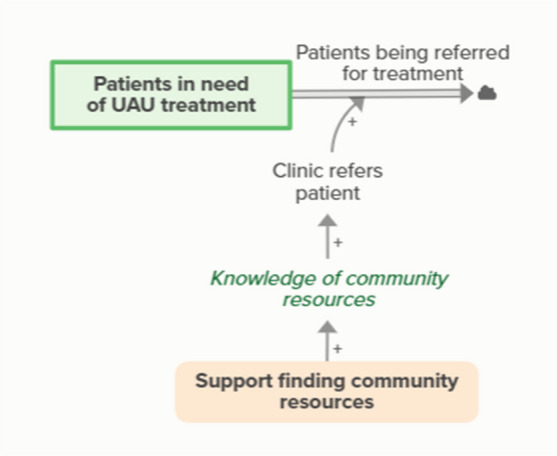	Facilitators supporting clinics in finding community resources improves clinic knowledge of community resources, which supports the ability of clinics to refer patients in need of UAU treatment to community providers. When patients are referred for treatment, the stock of people in need of UAU treatment but not yet referred decreases, forming an implicit feedback loop
Workflow mapping	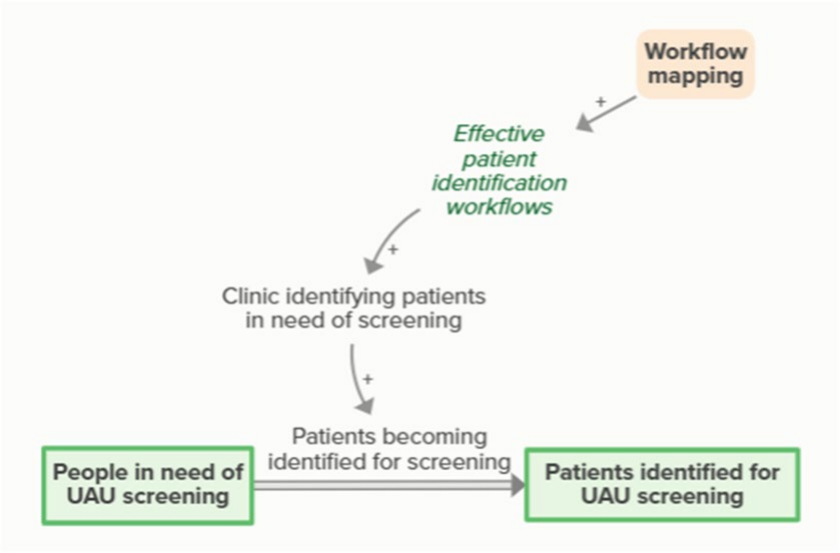	Workflow mapping enables effective patient identification workflows, which support clinics in identifying patients in need of screening. Identifying patients in need of screening means patients become identified for screening, which moves people from in need of screening to identified for screening. As more people become identified for UAU screening, fewer people are identified as in need of UAU screening, forming an implicit feedback loop
	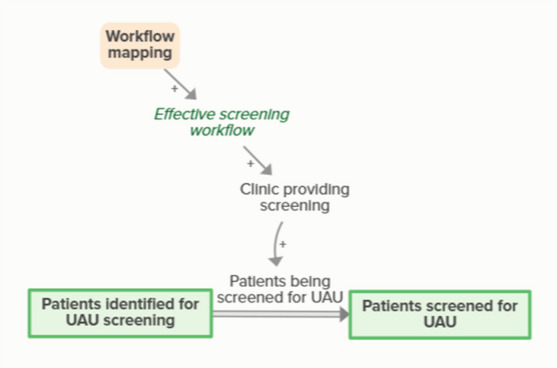	Workflow mapping enables effective screening workflows, which supports clinics in providing screening. Patients being screened for UAU moves people identified for screening to screened for UAU. As more patients are screened for UAU, fewer patients are identified for upcoming UAU screening, forming an implicit feedback loop
	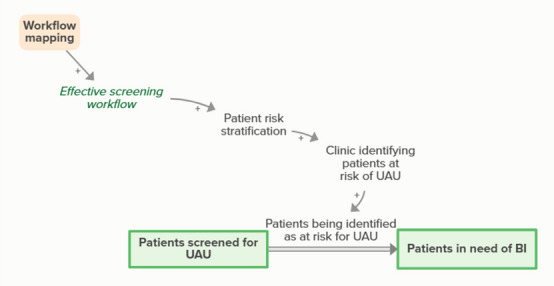	Workflow mapping enables effective BI workflows, which facilitates the clinic’s ability to identify and stratify patients’ risk for UAU. As more patients are screened and identified as at risk for UAU, the number of patients in need of BI increases and the number of patients screened for UAU but not yet identified as at risk decreases, forming an implicit feedback loop
	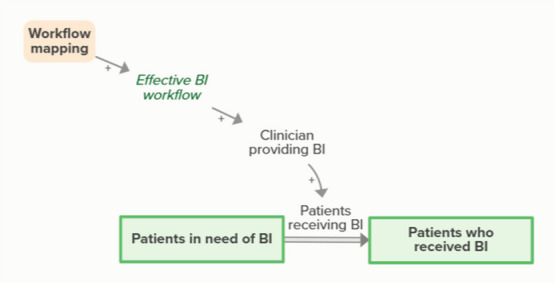	Workflow mapping enables effective BI workflows, which supports clinicians in providing BI. Clinicians providing and patients receiving BI moves people from in need of BI to received BI. As more patients receive BI, fewer patients are in need of BI, forming an implicit feedback loop
	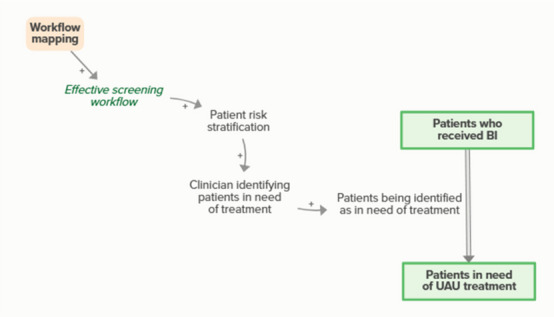	Workflow mapping enables effective screening workflows, which facilitates the clinic’s ability to identify and stratify patients’ risk for UAU and need for UAU treatment, increasing the number of patients identified as in need of treatment. As more patients are identified for UAU treatment, fewer patients are in the stock of patients who received BI but were not yet identified as in need of treatment, forming an implicit feedback loop.

The mechanisms detailed in Table 3 cover all feedback loops and flows in Fig. 3, with the exception of flows that indicate loss to follow up (e.g., *Patients declining or not provided screening*). Strategies were related to both patient care and QI components (shown in the rounded box in Fig. 3).

**Fig. 4:**
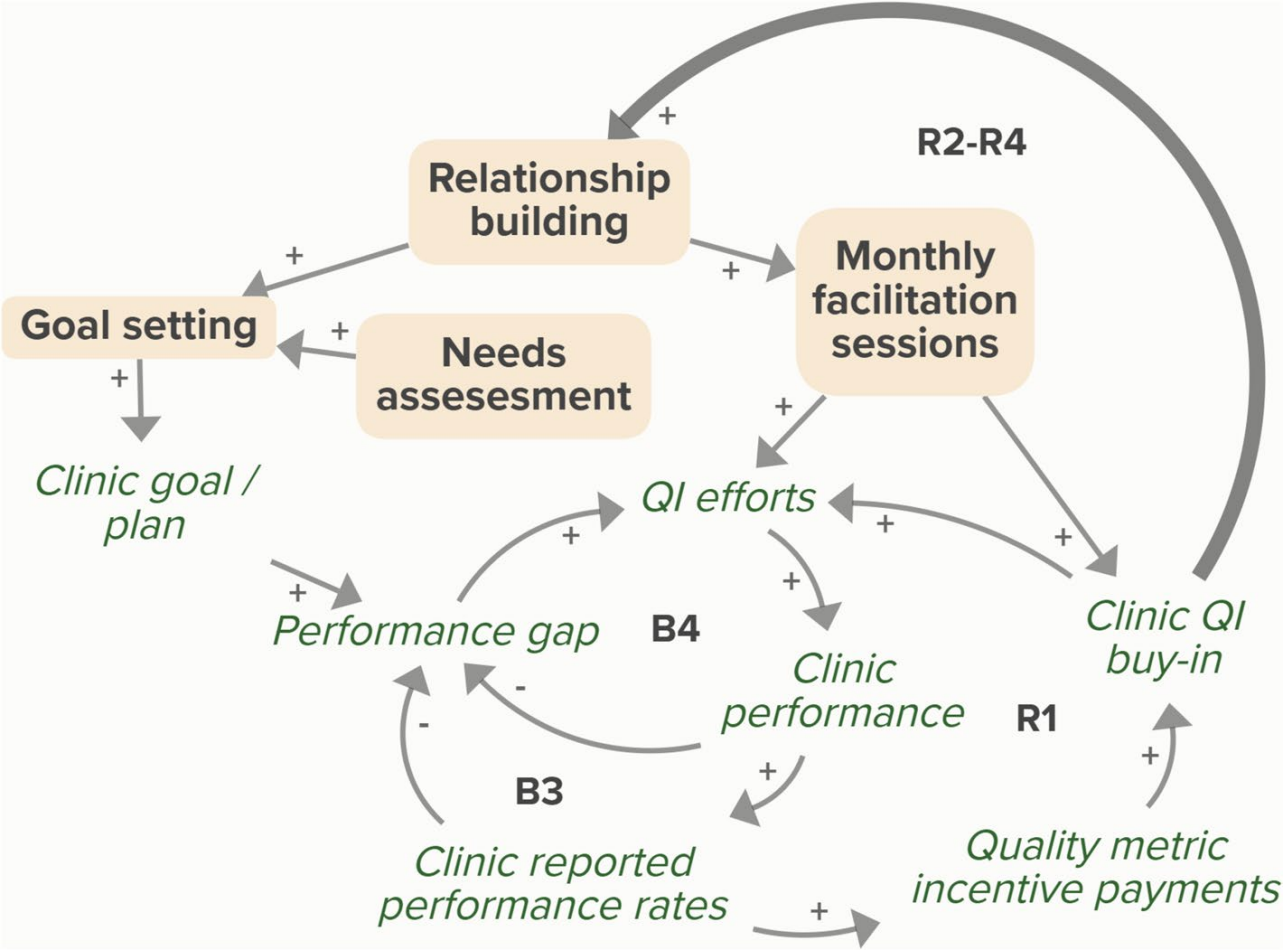
Secondary reinforcing feedback dynamics introduced in intervention. Over time, a facilitator’s activities to support clinic QI efforts (relationship building, goal setting, needs assessment, monthly facilitation sessions) result in increased clinic QI buy-in, which improves the clinic-facilitator relationship. Three new reinforcing loops (R2-R4) are introduced, indicating how efforts can be sustained over time. Arrows with a positive (+) or negative (-) valence communicate causal relationships between auxiliary variables. The thicker arrow indicates a new causal link. Balancing (B) and reinforcing (R) feedback loops are numbered

It should be noted that facilitators and clinics also described reinforcing dynamics in which a clinic’s positive experience with a facilitator leads to further buy-in and engagement in a strategy. Figure 4 shows how over time, increased clinic QI buy-in resulting from a facilitator’s efforts with a clinic lead to an improved facilitator-clinic relationship, which introduces new reinforcing feedback loops that help sustain the effort. Similar reinforcing dynamics could be described across any of the facilitator strategies. These dynamics can be seen on a longer time scale than the mechanisms described in Fig. 3 and Table 3 and can be understood as secondary to the primary mechanisms. For the sake of parsimony we chose to exclude these feedback loops from the intervention model in Fig. 3, but they should be considered when planning for implementation and sustainability.

## Discussion

By using system dynamics diagramming, an established systems science approach, we identified mechanisms by which practice facilitators supported implementation of SBIRT and MAUD in the ANTECEDENT study. In addition to informing future efforts to improve screening and treatment of UAU in primary care, this research contributes to the growing literature on practice facilitation and has implications for the study of mechanisms of implementation more broadly.

### Comparison to prior SBIRT modeling research

A similar study by Lounsbury and colleagues [62] utilized system dynamics modeling to simulate the effects of continuous technical assistance and site-specific performance feedback reporting to assess how these strategies impacted the implementation of SBIRT in primary care settings. Such simulation models can be useful for extrapolating anticipated effects of implementation strategies over time. Our present study used a diagrammatic approach to illustrate the mechanisms underlying practice facilitator-supported strategies for SBIRT and MAUD implementation in primary care.

### Practice facilitation for SBIRT and MAUD in primary care

The strategies used by ANTECEDENT practice facilitators largely aligned with best practices and known challenges for SBIRT and MAUD implementation outlined in prior research, such as goal setting [33, 63, 64], HIT support and electronic health record (EHR) integration of validated screening tools [34, 35, 63, 65, 66], workflow mapping and alignment of SBIRT with clinic processes [34, 35, 66, 67], guidance on motivational interviewing and brief interventions [66, 68–70], and academic detailing [64]. More broadly, prior literature has shown the utility of practice facilitators to support the generation and reporting of electronic clinical quality measures to accurately reflect the provision of quality health care services in primary care and aid in quality incentive metric payments [63, 71, 72].

This system dynamics diagramming approach could be used to guide future implementation of SBIRT and MAUD in primary care practices. Facilitators could either adapt the diagrams produced in this paper to suit local context or create their own diagrams, potentially as part of goal setting with clinics [33]. When used from the start of the study, system dynamics diagrams could serve as ‘theories of change’ [73, 74] or conceptual models to inform implementation and analysis. Specifying the mechanisms through which implementation strategies are supposed to affect outcomes would enable implementation teams to better anticipate unintended consequences and adapt to changing contexts [22].

### Systems-informed conceptualization of mechanisms

Implementation science research has seen an increasing interest in mechanisms underlying implementation strategies, including practice facilitation [16, 20, 21, 75, 76]. As mentioned in prior research, specifying the mechanisms of implementation strategies can help provide clarity, prevent mismatch between strategy and context, and gain generalizable knowledge about mechanisms associated with certain strategies [20]. In defining mechanisms as connections between strategies and feedback loops, we offer an approach to conceptualize mechanisms using system dynamics diagramming.

Several prior efforts have been made to diagram implementation mechanisms [16, 20, 77]. Lewis and colleagues [20] created causal pathway models that specify relationships between implementation strategies, moderators, preconditions, proximal outcomes, and distal outcomes in a simple linear format. These diagrams describe mechanisms associated with single implementation strategies and are intended to illustrate hypothesized causal relationships that can be tested experimentally. The authors are currently compiling a web-based reference database of strategy-mechanism linkages described with causal pathway models to support the selection of strategies to match specific contexts [78].

Causal pathway models can be contrasted with the mechanism diagrams we present in Table 3. Lewis’s causal pathway models maintain a rigid structure to maximize the distinction between types of factors in individual mechanisms and enable comparability between mechanisms. In our study, we used standard system dynamics diagramming notation, with the addition of distinguishing strategy variables. The most substantial difference between the two diagramming approaches is that our system dynamics diagramming approach describes mechanisms as including feedback loops, while the Lewis causal pathway models are linear and do not specify the valence of causal relationships. The inclusion of feedback loops (both explicit and implicit) is significant because feedback dynamics are responsible for nonlinear behavior in complex systems. Understanding these nonlinear patterns of behavior is important for understanding how systems change, a goal of the implementation mechanisms literature. System dynamics diagramming also allows for integration of individual mechanism diagrams into a composite diagram showing a dynamic hypothesis about how multiple strategies interact with contextual factors to produce outcomes. That being said, the two approaches are potentially compatible; system dynamics diagramming could be used to illustrate the complex dynamics of mechanisms in the broader context of interventions, while causal pathway models could be used to communicate the basic components of a mechanism across studies or contexts.

Our research can be compared to a recent study by Kilbourne and colleagues [16], which used a directed acyclic graph to describe the role of facilitation in healthcare. Like system dynamics diagrams, directed acyclic graphs are a type of diagram that describes hypothesized causal relationships between variables and outcomes [79, 80]. Directed acyclic graphs, however, describe linear pathways to outcomes and specifically do not include feedback loops. The graph created by Kilbourne and colleagues was intended to describe sequential mechanisms, or processes, of facilitation that apply across interventions and contexts. The variables included in their diagram, therefore, were largely relational factors (e.g., engaging leadership and champions). While directed acyclic graphs and system dynamics diagrams both describe hypothesized causal relationships, the methods have different purposes, strengths, and limitations. Because directed acyclic graphs do not contain feedback loops, they are well suited to describing linear sequences of events or changes in composition, such as versioning changes, genealogy, citation histories, or causes and effects of single variables [76, 81]. The inclusion of feedback loops enables system dynamics diagrams, particularly causal-loop diagrams, to describe how the relationships between variables in a system shape system behavior over time. Our approach also lends to the incorporation of important contextual factors, which heavily influences mechanisms (e.g. clinic resources, availability of screening tools, training, MOUD availability in clinic/community). By first illustrating how complex factors interact to produce problematic behavior in the usual care scenario, system dynamics diagrams can show the mechanisms by which interventions or implementation strategies act on those existing variables to change outcomes. In this way, system dynamics diagramming more transparently describes the mechanism of change in implementation strategies.

Many of the approaches to describe context or mechanisms of implementation serve to categorize and label factors or components. While categorization or classification is helpful for comparing across studies, it alone is insufficient for understanding change mechanisms [20], which by nature are dynamic and highly context dependent. When taking a systems science approach, distinctions between inner and outer context, or between interventions and implementation strategies, for example, are not in themselves important for understanding how systems change. What is important is how variables interact to shape dynamics that affect system behavior over time [30]. A systems approach involves developing an accurate dynamic hypothesis of how a problem in a specific context perpetuates over time and identifying effective points of leverage for changing that system to get the desired outcomes [30]. Any proposed change to a system (e.g., a policy, strategy, or intervention) is viewed in light of its anticipated impact to system structure and behavior. As implementation science expands its understanding of mechanisms of practice change, including practice facilitation, systems science will be valuable for ensuring that this understanding accounts for the complexity found in real-world implementation settings.

While we specify individual mechanisms in Table 3 to illustrate the ways in which strategies affect system behavior, it is important to emphasize that these mechanisms do not truly exist in isolation. A strength of the system dynamics diagramming approach is that it describes how the mechanisms are interconnected to each other and other components of the system. These unique interconnections defined by local context shape the system behavior or outcomes we see. For that reason, we do not see the individual mechanisms as necessarily universal across settings. The extent to which mechanisms are common across settings is an open question that can be investigated.

### Practice facilitators as complexity navigators

Our research demonstrates how practice facilitation is a highly iterative, context-sensitive meta-strategy. Figure 3 illustrates how practice facilitators used strategies that activated mechanisms related to every feedback loop in the usual care scenario, with the exception of ‘loss to follow-up’ outflows. This indicates that in a flexible, tailored implementation study, the facilitators identified strategies that addressed every opportunity for change in the implementation context. The specific strategies used by facilitators were customized to the needs of clinics as identified during the course of implementation and adjusted as those needs changed. Illustrating the mechanisms of practice facilitation as relating to a specific implementation project, therefore, does justice to the embedded nature of practice facilitation in context [82]. The use of system dynamics diagramming to specify these mechanisms enables precision in connecting context, strategies, and outcomes via the feedback structures driving nonlinear behavior [30].

Implementation settings are often complex and practice facilitators are charged with identifying strategies that simultaneously address needs and pressures at patient, clinic, health system, health plan, and community levels [61]. These needs and pressures are highly context dependent. Practice facilitators can therefore be understood to occupy the role of ‘complexity navigators,’ who seek to identify strategies that address multilevel contextual needs while adhering to core aspects of an intervention. This nuance of practice facilitators identifying strategies based on context also aligns with a recent study of practice facilitators’ tailoring efforts [44]. As practice facilitation increasingly becomes an established profession with its own body of expertise, the role of facilitators as complexity navigators can be considered [10, 83].

### Limitations

Our study has several limitations. While the application of system dynamics diagramming was suitable for describing the mechanisms of practice facilitation in the ANTECEDENT study, our research described only the single application. As described above, the generalizability of these mechanisms to other instances of SBIRT implementation or other interventions is unknown. Because our analysis was based on a diagram rather than an operational simulation model, our findings were limited to describing mechanisms of the intervention as understood by the study team. A simulation model based on observational data about patient flows would enable comparison of the degree of influence of mechanisms individually or in combination. It is also important to note that the practice facilitators in our study were also new to facilitation. It is possible that we would have seen different results with more experienced facilitators. We also experienced a substantial amount of staff turnover among practice facilitators, with none of the initial facilitators present by the study’s close-out. Finally, the study was conducted concurrently with the COVID-19 pandemic, which necessitated a shift to virtual facilitation meetings and introduced significant external pressure, particularly on primary care clinics and on research teams working in these settings [84]. These external pressures likely shaped the strategies used by practice facilitators, and therefore the mechanisms identified in our study.

### Future research

Future research could examine whether the facilitator strategies and associated mechanisms identified in this study are also seen in other SBIRT implementation efforts. Future research should also examine the applicability of this diagramming approach to other interventions and implementation contexts that utilize practice facilitation. As a body of research emerges, recommendations for best practice could provide guidance for the use of system dynamics diagramming to describe mechanisms in implementation science. Future scholarship could seek to further compare or align this diagramming approach with other approaches to describing causal mechanisms, such as causal pathway models. This approach could also be expanded to examine adaptation of implementation strategies and refine our understanding of implementation strategies as bundles of activities versus individual behaviors [39].

## Conclusions

System dynamics diagramming can be used to identify and illustrate mechanisms underlying strategies used by practice facilitators in a pragmatic implementation study. Because this approach connects strategies with the complex dynamics found in specific implementation settings, it is well suited for describing the mechanisms by which strategies act on contexts to produce changes in outcomes. By applying this approach to the ANTECEDENT study, we showed how practice facilitators use a variety of strategies when engaging clinics in an flexible implementation study, including goal setting, relationship building, workflow assessment, and HIT support. The role of practice facilitators can be considered ‘complexity navigators’ in implementation deserves future investigation. Additionally, the use of system dynamics diagramming to illustrate mechanisms in implementation, including mechanisms associated with practice facilitation, should be further developed and tested.

## Supplementary Information

Supplementary Material 1.

Supplementary Material 2.

## Data Availability

The datasets used and/or analysed during the current study are available from the corresponding author on reasonable request.

## Abbreviations

ERIC: Expert Recommendations for Implementation Change
QI: Quality improvement
EBI: Evidence-based intervention
ANTECEDENT: Partnerships to Enhance Alcohol Screening, Treatment, and Intervention
UAU: Unhealthy alcohol use
IRB: Institutional Review Board
SBIRT: Screening, brief intervention, referral to treatment
MAUD: Medications for alcohol use disorder
BI: Brief intervention
ORPRN: Oregon Rural Practice-based Research Network
AHRQ: Agency for Healthcare Research and Quality
HIT: Health information technology
PDSA: Plan-do-study-act
MI: Motivational interviewing
ECHO: Oregon Extension for Community Health Outcomes
EHR: Electronic health record

## References

[1] Powell BJ, Waltz TJ, Chinman MJ, Damschroder LJ, Smith JL, Matthieu MM, et al. A refined compilation of implementation strategies: results from the Expert Recommendations for Implementing Change (ERIC) project. Implement Sci. 2015;10(1):21. 25889199 10.1186/s13012-015-0209-1 PMC4328074

[2] Harvey G, Loftus-Hills A, Rycroft-Malone J, Titchen A, Kitson A, McCormack B, et al. Getting evidence into practice: the role and function of facilitation. J Adv Nurs. 2002;37(6):577–88. 11879422 10.1046/j.1365-2648.2002.02126.x

[3] Stetler CB, Legro MW, Rycroft-Malone J, Bowman C, Curran G, Guihan M, et al. Role of “external facilitation” in implementation of research findings: a qualitative evaluation of facilitation experiences in the Veterans Health Administration. Implement Sci. 2006;18(1):23. 10.1186/1748-5908-1-23 PMC1635058 17049080

[4] Kitson A, Harvey G, McCormack B. Enabling the implementation of evidence based practice: a conceptual framework. Qual Health Care QHC. 1998;7(3):149–58. 10185141 10.1136/qshc.7.3.149 PMC2483604

[5] Baskerville NB, Liddy C, Hogg W. Systematic review and meta-analysis of practice facilitation within primary care settings. Ann Fam Med. 2012;10(1):63–74. 22230833 10.1370/afm.1312 PMC3262473

[6] Nguyen AM, Cuthel A, Padgett DK, Niles P, Rogers E, Pham-Singer H, et al. How practice facilitation strategies differ by practice context. J Gen Intern Med. 2020;35(3):824–31. 31637651 10.1007/s11606-019-05350-7 PMC7080927

[7] Ashcraft LE, Goodrich DE, Hero J, Phares A, Bachrach RL, Quinn DA, et al. A systematic review of experimentally tested implementation strategies across health and human service settings: evidence from 2010–2022. Implement Sci. 2024;19(1):43. 38915102 10.1186/s13012-024-01369-5 PMC11194895

[8] Berta W, Cranley L, Dearing JW, Dogherty EJ, Squires JE, Estabrooks CA. Why (we think) facilitation works: insights from organizational learning theory. Implement Sci. 2015;10(1):141. 26443999 10.1186/s13012-015-0323-0 PMC4596304

[9] Cole AM, Keppel GA, Baldwin LM, Holden E, Parchman M. Implementation strategies used by facilitators to improve control of cardiovascular risk factors in primary care. J Am Board Fam Med JABFM. 2024;37(3):444–54. 38942445 10.3122/jabfm.2023.230312R1 PMC11514367

[10] Ritchie MJ, Parker LE, Kirchner JE. Facilitating implementation of primary care mental health over time and across organizational contexts: a qualitative study of role and process. BMC Health Serv Res. 2023;1(23):565. 10.1186/s12913-023-09598-y PMC10233920 37259064

[11] Walunas TL, Ye J, Bannon J, Wang A, Kho AN, Smith JD, et al. Does coaching matter? Examining the impact of specific practice facilitation strategies on implementation of quality improvement interventions in the Healthy Hearts in the Heartland study. Implement Sci. 2021;16(1):33. 33789696 10.1186/s13012-021-01100-8 PMC8011080

[12] Curran GM, Bauer M, Mittman B, Pyne JM, Stetler C. Effectiveness-implementation hybrid designs. Med Care. 2012;50(3):217–26. 22310560 10.1097/MLR.0b013e3182408812 PMC3731143

[13] Lee AK, Bobb JF, Richards JE, Achtmeyer CE, Ludman E, Oliver M, et al. Integrating alcohol-related prevention and treatment into primary care: a cluster randomized implementation trial. JAMA Intern Med. 2023;183(4):319–28. 36848119 10.1001/jamainternmed.2022.7083 PMC9972247

[14] McNeely J, Adam A, Rotrosen J, Wakeman SE, Wilens TE, Kannry J, et al. Comparison of methods for alcohol and drug screening in primary care clinics. JAMA Netw Open. 2021;4(5):e2110721. 34014326 10.1001/jamanetworkopen.2021.10721 PMC8138691

[15] Perry CK, Damschroder LJ, Hemler JR, Woodson TT, Ono SS, Cohen DJ. Specifying and comparing implementation strategies across seven large implementation interventions: a practical application of theory. Implement Sci. 2019;14(1):32. 30898133 10.1186/s13012-019-0876-4 PMC6429753

[16] Kilbourne AM, Geng E, Eshun-Wilson I, Sweeney S, Shelley D, Cohen DJ, et al. How does facilitation in healthcare work? Using mechanism mapping to illuminate the black box of a meta-implementation strategy. Implement Sci Commun. 2023;4(1):53. 37194084 10.1186/s43058-023-00435-1 PMC10190070

[17] Sweeney SM, Baron A, Hall JD, Ezekiel-Herrera D, Springer R, Ward RL, et al. Effective facilitator strategies for supporting primary care practice change: a mixed methods study. Ann Fam Med. 2022;20(5):414–22. 36228060 10.1370/afm.2847 PMC9512557

[18] Wang A, Pollack T, Kadziel LA, Ross SM, McHugh M, Jordan N, et al. Impact of practice facilitation in primary care on chronic disease care processes and outcomes: a systematic review. J Gen Intern Med. 2018;33(11):1968–77. 30066117 10.1007/s11606-018-4581-9 PMC6206351

[19] Lewis CC, Powell BJ, Brewer SK, Nguyen AM, Schriger SH, Vejnoska SF, et al. Advancing mechanisms of implementation to accelerate sustainable evidence-based practice integration: protocol for generating a research agenda. BMJ Open. 2021;11(10):e053474. 34663668 10.1136/bmjopen-2021-053474 PMC8524292

[20] Lewis CC, Klasnja P, Powell BJ, Lyon AR, Tuzzio L, Jones S, et al. From classification to causality: advancing understanding of mechanisms of change in implementation science. Front Public Health. 2018;6:136. 29868544 10.3389/fpubh.2018.00136 PMC5949843

[21] Lewis CC, Boyd MR, Walsh-Bailey C, Lyon AR, Beidas R, Mittman B, et al. A systematic review of empirical studies examining mechanisms of implementation in health. Implement Sci IS. 2020 16;15(1):21. 10.1186/s13012-020-00983-3 PMC7164241 32299461

[22] Moore TR, Chusan YAC, Pachucki M, Kim B. A participatory systems approach for visualizing and testing implementation strategies and mechanisms: evidence adoption in community coalitions. Implement Sci Commun. 2025;6(1):96. 41035014 10.1186/s43058-025-00788-9 PMC12487054

[23] Vejnoska SF, Mettert K, Lewis CC. Mechanisms of implementation: an appraisal of causal pathways presented at the 5th biennial Society for Implementation Research Collaboration (SIRC) conference. Implement Res Pract. 2022;4(3):26334895221086270. 10.1177/26334895221086271 PMC9924245 37091081

[24] Geng EH, Powell BJ, Goss CW, Lewis CC, Sales AE, Kim B. When the parts are greater than the whole: how understanding mechanisms can advance implementation research. Implement Sci. 2025;20(1):22. 40361178 10.1186/s13012-025-01427-6 PMC12070568

[25] Kazdin AE. Mediators and mechanisms of change in psychotherapy research. Annu Rev Clin Psychol. 2007;3(1):1–27. 17716046 10.1146/annurev.clinpsy.3.022806.091432

[26] Klasnja P, Meza RD, Pullmann MD, Mettert KD, Hawkes R, Palazzo L, et al. Getting cozy with causality: Advances to the causal pathway diagramming method to enhance implementation precision. Implement Res Pract. 2024;1(5):26334895241248852. 10.1177/26334895241248851 PMC11062231 38694167

[27] Kim B, Cruden G, Crable EL, Quanbeck A, Mittman BS, Wagner AD. A structured approach to applying systems analysis methods for examining implementation mechanisms. Implement Sci Commun. 2023;4(1):127. 37858215 10.1186/s43058-023-00504-5 PMC10588196

[28] Luke DA, Morshed AB, McKay VR, Combs TB. Systems Science Methods in Dissemination and Implementation Research. In: Dissemination and Implementation Research in Health. 2nd ed. New York: Oxford University Press; 2017. Available from: https://oxford.universitypressscholarship.com/10.1093/oso/9780190683214.001.0001/oso-9780190683214-chapter-10. Cited 2022 July 1.

[29] Forrester JW. Some basic concepts in system dynamics. Sloan Sch Manag Mass Inst Technol. 2009;29:17.

[30] Sterman J. Business Dynamics: Systems Thinking and Modeling for a Complex World. Irwin/McGraw-Hill; 2000. 982 p.

[31] Singh AN, Sanchez V, Kenzie ES, Sullivan E, McCormack JL, Larson JH, et al. Improving screening, treatment, and intervention for unhealthy alcohol use in primary care through clinic, practice-based research network, and health plan partnerships: protocol of the ANTECEDENT study. PLoS One. 2022;17(6):e0269635. 35763485 10.1371/journal.pone.0269635 PMC9239445

[32] Davis MM, Coury J, Sanchez V, Kenzie ES, Hiebert Larson J, Barnes C, et al. Improving screening, brief intervention and referral to treatment for unhealthy alcohol use in diverse, low-resourced primary care clinics. BMC Health Serv Res. 2024;24(1):1384. 39533319 10.1186/s12913-024-11870-8 PMC11556179

[33] Kenzie ES, Weekley T, Barnes C, Seater M, Sánchez V, Hatch BA, et al. Transl Behav Med. 2024;22:ibae059. 10.1093/tbm/ibae059 39438254

[34] Hargraves D, White C, Frederick R, Cinibulk M, Peters M, Young A, et al. Implementing SBIRT (Screening, Brief Intervention and Referral to Treatment) in primary care: lessons learned from a multi-practice evaluation portfolio. Public Health Rev. 2017;29(38):31. 10.1186/s40985-017-0077-0 PMC5809898 29450101

[35] Muench J, Jarvis K, Gray M, Hayes M, Vandersloot D, Hardman J, et al. Implementing a team-based SBIRT model in primary care clinics. J Subst Use. 2015;20(2):106–12.

[36] Kenzie ES, Wakeland W, Jetter A, Lich KH, Seater M, Davis MM. Mapping mental models through animproved method for identifying causal structures in qualitative data. Syst Res Behav Sci. 2025;42(3):756–71.

[37] Kenzie ES, Campbell J, Seater M, Singh MA, Robbins A, Davis MM. Goal alignment and unintended consequences of accountable care: How the structure of Oregon’s Medicaid coordinated care model shapes health plan–clinic partnerships. J Clin Transl Sci. 2025;9(1):e46. 10.1017/cts.2025.26 PMC11975777 40201654

[38] Holland JH. Studying complex adaptive systems. J Syst Sci Complex. 2006;19(1):1–8.

[39] Davis MM, Gunn R, Kenzie E, Dickinson C, Conway C, Chau A, et al. Integration of improvement and implementation science in practice-based research networks: a longitudinal, comparative case study. J Gen Intern Med. 2021;36(6):1503–13. 33852140 10.1007/s11606-021-06610-1 PMC8175491

[40] Fagnan LJ, Morris C, Shipman SA, Holub J, King A, Angier H. Characterizing a practice-based research network: Oregon Rural Practice-Based Research Network (ORPRN) survey tools. J Am Board Fam Med. 2007;20(2):204–19. 17341758 10.3122/jabfm.2007.02.060140

[41] AHRQ. EvidenceNOW: Managing Unhealthy Alcohol Use | The Academy. Available from: https://integrationacademy.ahrq.gov/about/initiatives/alcohol. Cited 2025 Jan 28.

[42] Oregon Health & Science University Department of Family Medicine. SBIRT Oregon. Available from: http://www.sbirtoregon.org. Cited 2025 Jan 28.

[43] Weeks MR, Lounsbury DW, Li J, Hirsch G, Berman M, Green HD, et al. Simulating system dynamics of the HIV care continuum to achieve treatment as prevention. PLoS One. 2020;15(3):e0230568. 32191771 10.1371/journal.pone.0230568 PMC7082036

[44] Barnes C, Kenzie ES, Thomas T, Weekley T, Sanchez V, Hatch BA, et al. Tailoring implementation strategies to primary care clinic contexts through practice facilitation: Lessons learned from the ANTECEDENT study. Implement Sci Commun. 2026. 10.1186/s43058-026-00895-1. 10.1186/s43058-026-00895-1 PMC13067476 41782073

[45] Kenzie ES, Weekley T, Barnes C, Seater M, Sánchez V, Hatch BA, Coury J, Davis MM. Co-created improvement goals and strategies for implementing SBIRT and MAUD in primary care settings in a facilitator-supported, tailored implementation study. Transl Behav Med. 2025;15(1):ibae059. 10.1093/tbm/ibae059 39438254

[46] McCormack JL, Thomas TL, Barnes C, Sanchez V, Kenzie ES, Coury J, Davis MM. Challenges using electronic health records to support unhealthy alcohol use screening and intervention in primary care practices in the Pacific Northwest. J Am Med Inform Assoc. 2025;32(7):1157–63. 10.1093/jamia/ocaf083 PMC12198768 40478440

[47] Kenzie ES, Seater M, Wakeland W, Coronado GD, Davis MM. System dynamics modeling for cancer prevention and control: a systematic review. PLoS One. 2023;18(12):e0294912. 38039316 10.1371/journal.pone.0294912 PMC10691687

[48] Homer JB, Hirsch GB. System dynamics modeling for public health: background and opportunities. Am J Public Health. 2006;96(3):452–8. 16449591 10.2105/AJPH.2005.062059 PMC1470525

[49] Jadeja N, Zhu NJ, Lebcir RM, Sassi F, Holmes A, Ahmad R. Using system dynamics modelling to assess the economic efficiency of innovations in the public sector - a systematic review. PLoS One. 2022;17(2):e0263299. 35143541 10.1371/journal.pone.0263299 PMC8830692

[50] Jalali MS, Rahmandad H, Bullock SL, Ammerman A. Dynamics of implementation and maintenance of organizational health interventions. Int J Environ Res Public Health. 2017;14(8):917. 28809807 10.3390/ijerph14080917 PMC5580620

[51] Liu S, Osgood N, Gao Q, Xue H, Wang Y. Systems simulation model for assessing the sustainability and synergistic impacts of sugar-sweetened beverages tax and revenue recycling on childhood obesity prevention. J Oper Res Soc. 2016;67(5):708–21.

[52] Kenzie ES, Wakeland W, Jetter A, Hassmiller Lich K, Seater M, Davis MM. Mapping feedback dynamics in mental models through an improved method for identifying causal structures in qualitative data. Syst Res Behav Sci. 2023;42(3):756–71.

[53] Vanderby S, Carter MW. An evaluation of the applicability of system dynamics to patient flow modelling. J Oper Res Soc. 2010;61(11):1572–81.

[54] Lane DC, Husemann E. System dynamics mapping of acute patient flows. J Oper Res Soc. 2008;59(2):213–24.

[55] Davahli MR, Karwowski W, Taiar R. A system dynamics simulation applied to healthcare: a systematic review. Int J Environ Res Public Health. 2020;17(16):5741. 32784439 10.3390/ijerph17165741 PMC7460395

[56] Liang B, Turkcan A, Ceyhan ME, Stuart K. Improvement of chemotherapy patient flow and scheduling in an outpatient oncology clinic. Int J Prod Res. 2015;53(24):7177–90.

[57] Lindberg J, Holmström P, Hallberg S, Björk-Eriksson T, Olsson CE. An analytical approach to aggregate patient inflows to a simulation model over the radiotherapy process. BMC Health Serv Res. 2021;21(1):207. 33685475 10.1186/s12913-021-06162-4 PMC7938525

[58] Kenzie E, Seater M, Badicke B, Coury J, Coronado GD, Davis MM. Identification of feedback dynamics underlying colorectal cancer screening interventions using system dynamics diagramming. In: 17th Annual Dissemination and Implementation Conference. Arlington, Virginia, USA; 2024.

[59] Hovmand PS, Andersen DF, Rouwette E, Richardson GP, Rux K, Calhoun A. Group Model‐Building ‘Scripts’ as a Collaborative Planning Tool. Systems Research and Behavioral Science. 2012;29(2):179–93.

[60] Kumu. Available from: https://www.kumu.io/. Cited 2023 May 16.

[61] Nguyen AM, Cuthel A, Padgett DK, Niles P, Rogers E, Pham-Singer H, et al. How practice facilitation strategies differ by practice context. J Gen Intern Med. 2020;35(3):824–31. 31637651 10.1007/s11606-019-05350-7 PMC7080927

[62] Lounsbury DW, Mitchell SG, Dusek KA, Li JZ, Kirk AS, Oros M, et al. Application of system dynamics to inform a model of adolescent SBIRT implementation in primary care settings. J Behav Health Serv Res. 2020;47(2):230–44.31214935 10.1007/s11414-019-09650-y

[63] Hemler JR, Hall JD, Cholan RA, Crabtree BF, Damschroder LJ, Solberg LI, et al. Practice facilitator strategies for addressing electronic health record data challenges for quality improvement: evidenceNOW. J Am Board Fam Med. 2018;31(3):398–409. 29743223 10.3122/jabfm.2018.03.170274 PMC5972525

[64] Frost MC, Ioannou GN, Tsui JI, Edelman EJ, Weiner BJ, Fletcher OV, et al. Practice facilitation to implement alcohol-related care in Veterans Health Administration liver clinics: a study protocol. Implement Sci Commun. 2020;31(1):68. 10.1186/s43058-020-00062-0 PMC7393339 32835226

[65] McNeely J, Hamilton L. Screening for unhealthy alcohol and drug use in general medicine settings. Med Clin North Am. 2022;106(1):13–28. 34823726 10.1016/j.mcna.2021.08.002

[66] Whitlock EP, Polen MR, Green CA, Orleans T, Klein J. Behavioral counseling interventions in primary care to reduce risky/harmful alcohol use by adults: a summary of the evidence for the U.S. preventive services task force. Ann Intern Med. 2004;140(7):557. 15068985 10.7326/0003-4819-140-7-200404060-00017

[67] Huffstetler AN, Kuzel AJ, Sabo RT, Richards A, Brooks EM, Lail Kashiri P, et al. Practice facilitation to promote evidence-based screening and management of unhealthy alcohol use in primary care: a practice-level randomized controlled trial. BMC Fam Pract. 2020;21(1):93. 32434467 10.1186/s12875-020-01147-4 PMC7240919

[68] US Preventive Services Task Force. Screening and behavioral counseling interventions to reduce unhealthy alcohol use in adolescents and adults: US preventive services task force recommendation statement. JAMA. 2018;320(18):1899–909. 30422199 10.1001/jama.2018.16789

[69] Manuel JK, Satre DD, Tsoh J, Moreno-John G, Ramos JS, McCance-Katz EF, et al. Adapting Screening, Brief Intervention and Referral to Treatment (SBIRT) for alcohol and drugs to culturally diverse clinical populations. J Addict Med. 2015;9(5):343–51. 26428359 10.1097/ADM.0000000000000150 PMC4626638

[70] Babor TF, Higgins-Biddle JC, Dauser D, Burleson JA, Zarkin GA, Bray J. Brief interventions for at-risk drinking: patient outcomes and cost-effectiveness in managed care organizations. Alcohol Alcohol. 2006;41(6):624–31. 17035245 10.1093/alcalc/agl078

[71] Richardson JE, Rasmussen LV, Dorr DA, Sirkin JT, Shelley D, Rivera A, et al. Generating and reporting electronic clinical quality measures from electronic health records: strategies from evidenceNOW cooperatives. Appl Clin Inform. 2022;13(2):485–94. 35508198 10.1055/s-0042-1748145 PMC9068273

[72] Knierim KE, Hall TL, Dickinson LM, Nease DE, de la Cerda DR, Fernald D, et al. Primary care practices’ ability to report electronic clinical quality measures in the evidenceNOW Southwest initiative to improve heart health. JAMA Netw Open. 2019;2(8):e198569. 31390033 10.1001/jamanetworkopen.2019.8569 PMC6687038

[73] Kenzie ES. Get your model out there: Advancing methods for developing and using causal-loop diagrams [Dissertation]. Portland State University; 2021. Available from: https://pdxscholar.library.pdx.edu/open_access_etds/5664/.

[74] Hassmiller Lich K, Urban JB, Frerichs L, Dave G. Extending systems thinking in planning and evaluation using group concept mapping and system dynamics to tackle complex problems. Eval Program Plann. 2017;1(60):254–64. 10.1016/j.evalprogplan.2016.10.008 27825622

[75] Aldridge WA, Roppolo RH, Brown J, Bumbarger BK, Boothroyd RI. Mechanisms of change in external implementation support: a conceptual model and case examples to guide research and practice. Implement Res Pract. 2023;1(4):26334895231179760. 10.1177/26334895231179761 PMC10291867 37790181

[76] Geng EH, Baumann AA, Powell BJ. Mechanism mapping to advance research on implementation strategies. PLoS Med. 2022;19(2):e1003918. 35134069 10.1371/journal.pmed.1003918 PMC8824331

[77] Ike B, Johnson A, Meza R, Cole A. Integrating causal pathway diagrams into practice facilitation to address colorectal cancer screening disparities in primary care. BMC Health Serv Res. 2024;24(1):1007. 39215282 10.1186/s12913-024-11471-5 PMC11365243

[78] Lewis CC, Klasnja P, Lyon AR, Powell BJ, Lengnick-Hall R, Buchanan G, et al. The mechanics of implementation strategies and measures: advancing the study of implementation mechanisms. Implement Sci Commun. 2022;3(1):114. 36273224 10.1186/s43058-022-00358-3 PMC9588220

[79] Digitale JC, Martin JN, Glymour MM. Tutorial on directed acyclic graphs. J Clin Epidemiol. 2022;1(142):264–7. 10.1016/j.jclinepi.2021.08.001 PMC8821727 34371103

[80] Byeon S, Lee W. Directed acyclic graphs for clinical research: a tutorial. J Minim Invasive Surg. 2023;26(3):97–107. 37712307 10.7602/jmis.2023.26.3.97 PMC10505364

[81] Rodrigues D, Kreif N, Lawrence-Jones A, Barahona M, Mayer E. Reflection on modern methods: constructing directed acyclic graphs (DAGs) with domain experts for health services research. Int J Epidemiol. 2022;51(4):1339–48. 35713577 10.1093/ije/dyac135 PMC9365627

[82] Nagykaldi Z, Mold JW, Aspy CB. Practice facilitators: a review of the literature. Fam Med. 2005;37(8):581–8. 16145629

[83] Badicke B, Davis MM, Nagykaldi Z, Lipman PD, Dluzak L, Haddad L. Fifth international conference on practice facilitation (ICPF) focuses on building resilience and promoting professional wellness. Ann Fam Med. 2023;21(1):95–6.

[84] Holtrop JS, Davis MM. Primary care research is hard to do during COVID-19: challenges and solutions. Ann Fam Med. 2022;20(6):568–72. 36443077 10.1370/afm.2889 PMC9705050

