# Supplementary material for: Mechanisms of practice facilitation identified using system dynamics diagramming in a tailored implementation study of unhealthy alcohol screening and treatment in primary care
Source: Implement Sci Commun. 2026 Apr 13;7:100. doi: 10.1186/s43058-026-00909-y (PMC13191852; doi:10.1186/s43058-026-00909-y)
Supplement: Supplementary file 1 — Supplementary Material 1. [file 43058_2026_909_MOESM1_ESM.docx]

**Additional File 1. Sample interview questions for qualitative data collection**

From a guide for semi-structured interviews conducted with practice facilitators:

- How would you describe the experience of getting data from the clinics?
  - How was the data collection process adapted to fit the clinic context?
- How was ANTECEDENT implementation support tailored (either by study team, facilitators, or clinics) to fit local context and needs?
- Which implementation strategies were most effective for making changes in SBIRT, MAT, or QI capacity at the clinic?
- How did implementation support relate to changes in SBIRT or QI capacity for clinics?
- What factors do you think promote successful change?
  - What do you feel like are inhibitors of practices changing?

From a periodic reflection interview guide:

- What are your thoughts on the importance or impact of relationship building between the clinic and [practice facilitator]? Have your views changed though the course of the project?

From semi-structured interviews conducted with clinic staff at end of study:

- The ANTECEDENT study involved support for improve screening, brief intervention, and referral to treatment, or SBIRT, and the use of MAT for unhealthy alcohol use. Can you tell me about the type of improvement goals you set at the beginning of this study?
  - Why did you choose to focus on these goals?
  - Did your goals change during the study?
  - To what extent do you feel you were successful in achieving your goals?
- What kind of support did you receive from our project team?
  - Did the support you receive meet your needs? How or how not?
  - Could you talk about what was helpful or challenging related to the support you received?
  - Are there other resources/support that would help you in the future?
