# Supplementary material for: Mechanisms of practice facilitation identified using system dynamics diagramming in a tailored implementation study of unhealthy alcohol screening and treatment in primary care
Source: Implement Sci Commun. 2026 Apr 13;7:100. doi: 10.1186/s43058-026-00909-y (PMC13191852; doi:10.1186/s43058-026-00909-y)
Supplement: Supplementary file 2 — Supplementary Material 2. [file 43058_2026_909_MOESM2_ESM.docx]

Additional file 2. Codebook for secondary coding of qualitative data

| **Code** | **Definition** |
| --- | --- |
| @Needs Discussion | Process code; text needs discussion among analysts to clarify |
| Academic detailing | Use of faculty expert consultations and educational outreach to improve provider knowledge |
| Clinician and staff buy-in/motivation/engagement | Support or engagement by clinicians and clinic staff |
| Competing clinic priorities | Factors that influenced clinics’ ability to engage in the study |
| Goal setting | Collaborative development of implementation goals |
| HIT support | Use of data experts to improve clinic’s HIT |
| Learning collaborative | Structured method for supporting practice change in which peers and experts learn from each other |
| Modeling_misc | Quotations deemed by the analyst to be relevant to the practice facilitator model but not otherwise coded |
| Motivational interviewing | Approach used by clinicians for brief intervention |
| Needs assessment | Identification of clinic-level factors and needs |
| PDSA cycles | Plan-do-study-act cycles, a QI approach used by facilitators |
| PF flexibility/adaptability | Extent to which facilitators are or need to be flexible or adaptive in their approach with clinics |
| PF skills/training _difficulties or improvement | Excerpts related to training and skills of practice facilitators |
| PF turnover/transitions | Staff turnover or transitions among practice facilitator staff |
| QI tools_other | Additional mentions of QI tools not otherwise specified in the codebook |
| Relationship building | Building trust and cultivating relationships with clinical partners |
| Resource sharing | Sharing of relevant resources |
| Supporting accountability/clinic ownership | Actions taken to support clinic staff’s engagement and ownership over the change process |
| Training/education | General training and education to clinics |
| Troubleshooting | Ad hoc support provided to clinics |
| Workflow mapping | Diagramming of clinical workflows |
